# Neurotrophic factors and target-specific retrograde signaling interactions define the specificity of classical and neuropeptide cotransmitter release at identified *Lymnaea* synapses

**DOI:** 10.1038/s41598-020-70322-5

**Published:** 2020-08-11

**Authors:** Angela M. Getz, Tara A. Janes, Frank Visser, Wali Zaidi, Naweed I. Syed

**Affiliations:** 1 Department of Cell Biology and Anatomy, Hotchkiss Brain Institute, Alberta Children’s Hospital Research Institute, University of Calgary, Calgary, AB T2N 1N4 Canada; 2 Department of Neuroscience, Hotchkiss Brain Institute, University of Calgary, Calgary, AB T2N 1N4 Canada; 3 Department of Physiology and Pharmacology, Hotchkiss Brain Institute, University of Calgary, Calgary, AB T2N 1N4 Canada; 4 Present Address: Interdisciplinary Institute for Neuroscience, CNRS, UMR 5297, Université de Bordeaux, 33000 Bordeaux, France; 5 Present Address: Department of Physiology, Women and Children’s Health Research Institute, University of Alberta, Edmonton, T6G 1C9 AB AB Canada

**Keywords:** Cellular neuroscience, Neurotrophic factors, Synaptic transmission, Neurophysiology

## Abstract

Many neurons concurrently and/or differentially release multiple neurotransmitter substances to selectively modulate the activity of distinct postsynaptic targets within a network. However, the molecular mechanisms that produce synaptic heterogeneity by regulating the cotransmitter release characteristics of individual presynaptic terminals remain poorly defined. In particular, we know little about the regulation of neuropeptide corelease, despite the fact that they mediate synaptic transmission, plasticity and neuromodulation. Here, we report that an identified *Lymnaea* neuron selectively releases its classical small molecule and peptide neurotransmitters, acetylcholine and FMRFamide-derived neuropeptides, to differentially influence the activity of distinct postsynaptic targets that coordinate cardiorespiratory behaviour. Using a combination of electrophysiological, molecular, and pharmacological approaches, we found that neuropeptide cotransmitter release was regulated by cross-talk between extrinsic neurotrophic factor signaling and target-specific retrograde arachidonic acid signaling, which converged on modulation of glycogen synthase kinase 3. In this context, we identified a novel role for the *Lymnaea* synaptophysin homologue as a specific and synapse-delimited inhibitory regulator of peptide neurotransmitter release. This study is among the first to define the cellular and molecular mechanisms underlying the differential release of cotransmitter substances from individual presynaptic terminals, which allow for context-dependent tuning and plasticity of the synaptic networks underlying patterned motor behaviour.

## Introduction

Most neurons use more than one type of neurotransmitter to mediate synaptic transmission^1,2^. The use of multiple neurotransmitters enhances a neuron’s capacity to encode information within a circuit, allowing for more complex patterns of synaptic interactions to emerge, and the opportunity to differentially influence the activity of synaptic partners over multiple timescales^3^. Many neurons form hundreds of presynapses, innervate a variety of postsynaptic targets, and modulate their transmitter output in response to a wide range of intracellular and extracellular signals^4^. There is accumulating evidence that a neuron’s individual presynaptic terminals can exhibit distinct cotransmitter composition and release characteristics, and that this can be dynamically regulated in order to meet the spatiotemporal functional requirements of each synapse^5–13^. However, our understanding of the cellular and molecular mechanisms that allow a neuron to regulate the use of its cotransmitters at distinct synaptic sites is considerably lacking.

The concurrent use of classical small molecule and peptide neurotransmitters is common throughout the nervous system and across evolution. Neuropeptides are important modulatory signals that regulate numerous aspects of synaptic development, plasticity and network function, as well as animal physiology and behaviour^3,14^. Although peptide neurotransmitters are traditionally viewed as slow-acting neuromodulators released from perisynaptic sites in response to Ca^2+^ diffusion during high-frequency bursting^15^, their release has been demonstrated to occur over a wide range of firing frequencies^16–18^. Peptidergic large dense-core vesicles (LDCVs) have also been shown to cluster directly at presynaptic active zones and be released by single action potentials^19,20^. Furthermore, several studies have indicated that postsynaptic target identity influences the selective use of classical and peptide neurotransmitters at the different presynaptic terminals of a given neuron^6,19,21,22^. Taken together, these observations suggest that the peptidergic characteristics of cotransmitting terminals can be highly variable, although the molecular mechanisms that give rise to such differences are yet to be defined.

In this study we sought to identify the cellular and molecular mechanisms underlying presynaptic specificity, with a focus on two unresolved questions: (i) how does a neuron establish functionally distinct presynapses with specific postsynaptic targets, and (ii) how does a presynaptic neuron generate the appropriate patterns of cotransmitter release that are required for the coordinated actions of neurons in behaviourally-relevant networks? To address these outstanding questions, we used cardiorespiratory neurons from the central nervous system (CNS) of the invertebrate mollusc *Lymnaea stagnalis* to study how the selective use of cotransmitters at synapses with distinct postsynaptic targets influences the assembly and function of the neuronal circuits involved in cardiorespiratory regulation. Reductionist approaches using the simple nervous systems of invertebrate models have enabled fundamental insights into cotransmission because their large identified neurons with known transmitter phenotypes participate in behaviourally-defined synaptic networks that can be studied directly in situ or reconstructed and manipulated in vitro^23,24^. Moreover, invertebrate models are especially valuable for functional studies on peptidergic transmission because they are one of the few systems in which electrophysiologically measurable synaptic responses can be directly attributed to the actions of identified peptide neurotransmitters at individual synapses.

Here, we found that presynaptic neuropeptide release competency was regulated in a target- and context-dependent manner. This involved an interplay between extrinsic neurotrophic factors (NTF) and synapse-specific retrograde arachidonic acid (AA) signaling interactions, which converged on glycogen synthase kinase 3 (GSK-3) activity. In this context, we identified a surprising role for the *Lymnaea* synaptophysin (Syp) homologue in the inhibitory regulation of peptide neurotransmitter release. These findings on the regulation of cotransmitter use at individual synapses uncover a previously undefined mechanism for presynaptic cotransmitter specificity in synaptic networks, with implications for the appropriate expression and plasticity of patterned motor behaviours.

## Results

### Synaptic transmission via classical and peptide cotransmitters is target-specific

**Figure 1:**
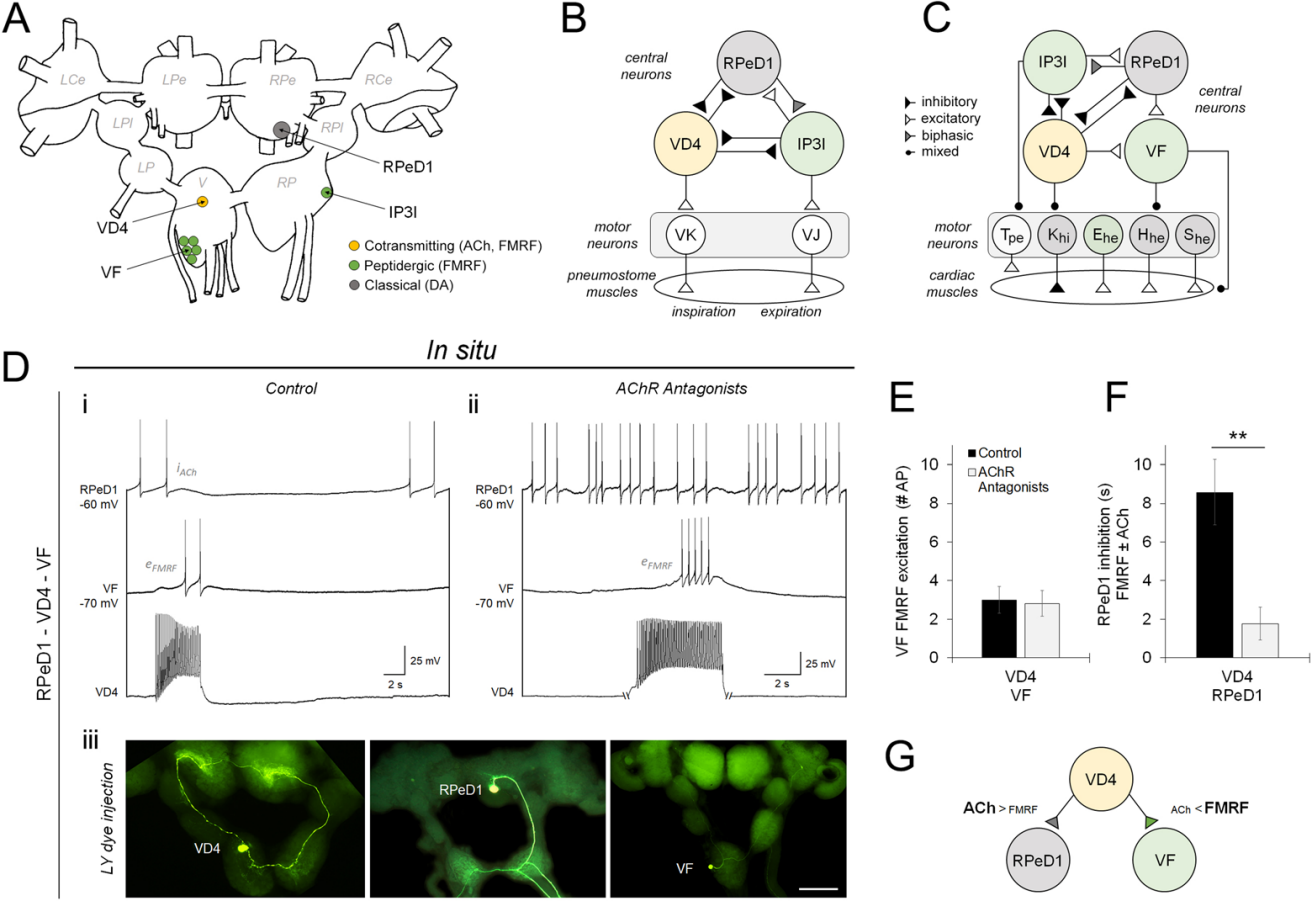
Synaptic transmission via classical and peptide cotransmitters is target-specific. (**A**). Schematic of the *Lymnaea* CNS, depicting in situ locations of the neurons used in this study. Dorsal surface of the ganglia: L/R, left and right; Ce, cerebral; Pe, pedal; Pl, pleural; P, parietal; V, visceral. Adapted from^23^. (**B**). Summary diagram of the neurons and synaptic connections that form the respiratory central pattern generator (rCPG) circuit, and the associated motor neurons that drive opening (expiration) and closing (inspiration) of the pneumostome. (**C**). Summary diagram of the cardioregulatory network, and the motor neurons implicated in modulating the rate and amplitude of auricular and ventricular contractions. (**D**). Simultaneous intracellular current clamp recordings of endogenous synapses from an isolated intact CNS preparation, performed in control conditions (**i**; *Lymnaea* normal saline), or with AChR antagonists (**ii**; 5 μM MLA, 10 μM TC, 20 μM TEA) to isolate peptidergic transmission. N = 11. Stimulation of VD4 elicits primarily cholinergic inhibition of RPeD1 (i_ACh_ in **i**, antagonized in **ii**) and primarily peptidergic excitation of VF (e_FMRF_ in **i** and **ii**). Lucifer yellow dye injections illustrate the morphology of identified cardiorespiratory neurons and their projections (**iii**). N ≥ 3 each. Scale bar, 1 mm. (**E**). Summary data, mean synaptic response of VF is unchanged by addition of AChR antagonists, indicating primarily peptidergic transmission (excitation of VF measured as number of action potentials induced in response to VD4 burst). *P* = 0.813 (Paired Samples T-test). (**F**). Summary data, mean synaptic response of RPeD1 is attenuated by AChR antagonists, indicating primarily cholinergic transmission (inhibition of RPeD1 measured as duration of hyperpolarization and firing cessation (s) induced in response to VD4 burst). **P = 0.003 (Wilcoxon Signed Ranks test). Error bars, SEM. (**G**). Summary diagram illustrating that synaptic transmission at distinct postsynaptic targets is differentially mediated by classical and peptide neurotransmitters.

To investigate the mechanisms that differentiate cotransmitter use at individual synapses, and the consequences of this for specifying network function and behaviour, we first sought to monitor synaptic transmission by classical and peptide transmitters at functionally-defined synapses. The *Lymnaea* cardiorespiratory interneuron visceral dorsal 4 (VD4) was of interest for this study as it is well known to use the classical small molecule neurotransmitter acetylcholine (ACh) alongside a mixture of neuropeptides derived from the heptapeptide transcript of the FMRFamide gene (primarily G/SDPFLRFamide; discussed in subsequent text as FMRF neuropeptides)^25–28^. VD4 is integrated into a three-neuron network via reciprocal inhibitory synapses with the FMRFamidergic input 3 interneuron (IP3I) and the giant dopaminergic neuron right pedal dorsal 1 (RPeD1). These neurons and their reciprocal synapses establish *Lymnaea*’s respiratory central pattern generator (rCPG) network in vivo, are indispensable for the expression of respiratory behaviour, and recapitulate appropriate synaptic networks and rhythmic activity when reconstructed in vitro^23,29^. VD4 is also known to form one-way excitatory synapses with a number of other identified neurons that act to coordinate cardiorespiratory behaviour, including the FMRFamidergic visceral F group cells (VF) and the serotonergic neuron left pedal dorsal 1 (LPeD1)^26,30–33^ (Fig. 1A–C). In *Lymnaea* neurons, both ACh and FMRF neuropeptides can elicit synaptic excitation or inhibition. For ACh, this is due to NTF-regulated expression of cationic or anionic nicotinic ACh receptors (AChR)^34–36^. For FMRF neuropeptides, this is due to cell type-specific coupling of FMRF G protein-coupled receptors (GPCRs) to distinct G protein complexes and metabotropic signaling cascades^37,38^. FMRF neuropeptides have also been shown to occlude transmitter release from target neurons by inhibitory regulation of Ca^2+^ influx and neurosecretory mechanisms^39^. We therefore wondered whether the release of ACh and FMRF neuropeptides might be selectively tuned at VD4′s presynaptic terminals to establish the target-specific formation of one-way or reciprocal, and inhibitory or excitatory synapses that are required for the appropriate expression and modulation of cardiorespiratory behaviour.

To investigate this question, we first performed simultaneous intracellular recordings from the isolated intact CNS to determine the classical vs peptidergic nature of synaptic transmission at endogenous synapses between VD4 and the identified cardiorespiratory neurons VF and RPeD1. Synaptic transmission was assessed in response to a ~ 4 s burst induced in VD4, first under control conditions to assay cotransmission, and then in the presence of an AChR antagonist cocktail (see “Methods” section) to isolate the peptidergic component of synaptic transmission. We found that excitatory transmission with VF was mediated primarily by FMRF neuropeptides, as the mean number of action potentials induced in VF in response to VD4 stimulation was unchanged after the addition of AChR antagonists. Inhibitory transmission with RPeD1, however, was mediated primarily by ACh, as the duration of RPeD1 inhibition in response to VD4 stimulation was reduced with AChR antagonists (Fig. 1D–F; Table S1). This suggests that synaptic transmission with distinct postsynaptic targets is not equivalently mediated by VD4′s classical and peptide cotransmitters (Fig. 1G).

### Target-specific regulation of cotransmission is modulated by extrinsic NTF signaling

Because the identified cardiorespiratory neurons and their synaptic networks cannot be easily resolved or manipulated in the intact brain preparation, we turned to in vitro reconstruction of these synapses to uncover the regulatory mechanisms that specify transmission by classical and peptide neurotransmitters. To this end, we isolated the identified neurons from the CNS and paired VD4 in a soma-soma configuration with RPeD1 or VF in culture. As in the intact preparation, we made simultaneous intracellular recordings from cultured neurons, first under control conditions to study cotransmission, and then in the presence of AChR antagonists to isolate peptidergic transmission.

**Figure 2:**
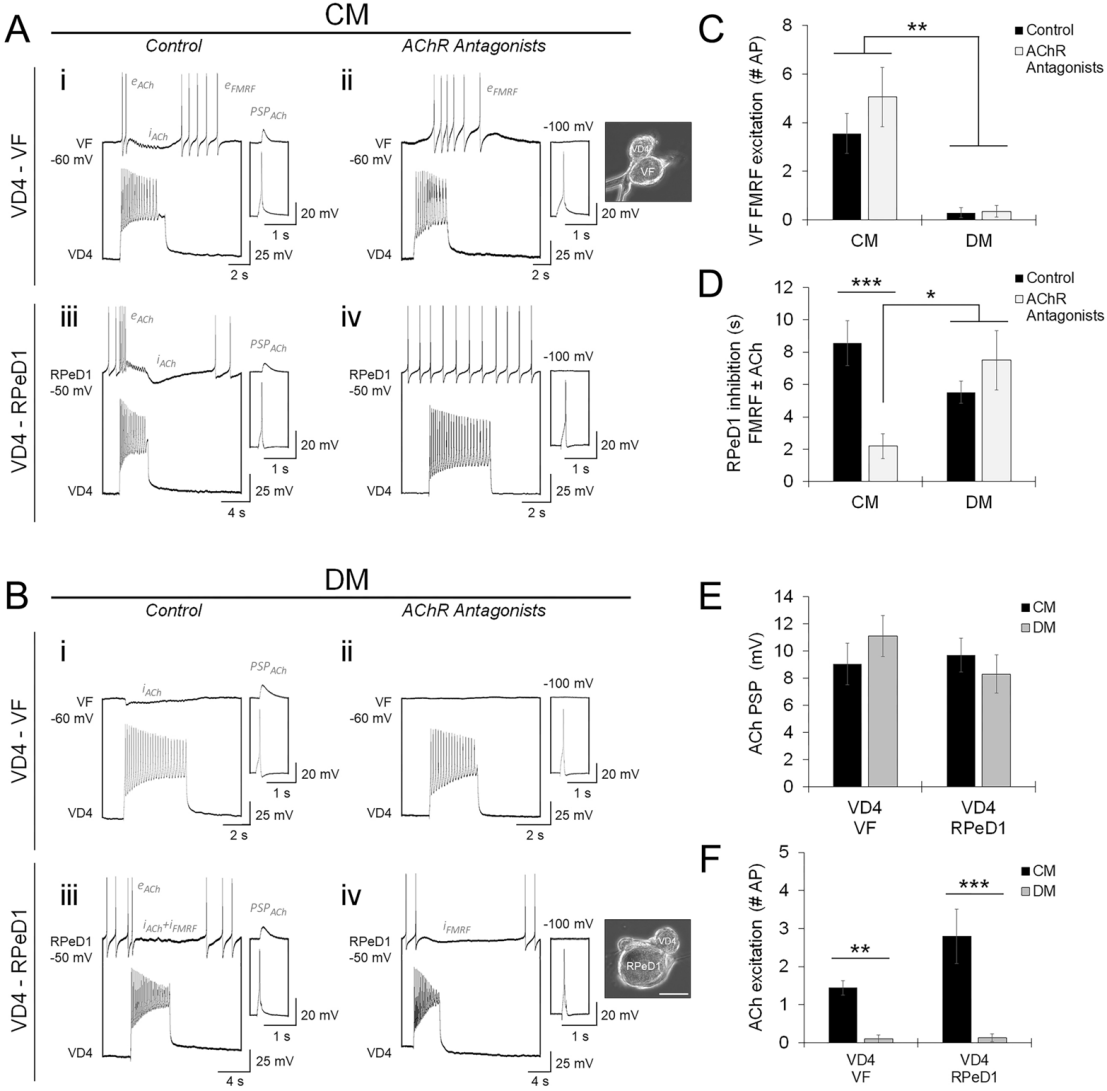
Target-specific use of cotransmitters is modulated by extrinsic neurotrophic factor signaling. (**A**–**B**). Characterization of cotransmission and isolated FMRF transmission by simultaneous intracellular current clamp recordings in soma-soma paired neurons (image inserts; scale bar, 50 μm), performed in CM or DM control (**Ai**,**iii** and **Bi**,**iii**), or with AChR antagonists (**Aii**,**iv** and **Bii**,**iv**; 5 μM MLA, 10 μM TC, 20 μM TEA) to isolate peptidergic transmission. Inserts show ACh-PSPs (**i**,**iii**; − 100 mV holding potential), inhibited by AChR antagonists (**ii**,**iv**). Mixed cholinergic-peptidergic cotransmission is observed at VD4-VF synapses in CM, with biphasic cholinergic and excitatory peptidergic responses (**Ai**,**ii**; e_ACh_, i_ACh_, e_FMRF_), and at VD4-RPeD1 synapses in DM, with biphasic cholinergic and inhibitory peptidergic responses (**Biii**,**iv**; e_ACh_, i_ACh_, i_FMRF_). Primarily cholinergic transmission is observed at VD4-RPeD1 synapses in CM (**Aiii**,**iv**; e_ACh_, i_ACh_, absence of i_FMRF_), and VD4-VF synapses in DM (**Bi**,**ii**; i_ACh_, absence of e_FMRF_), demonstrating that peptide cotransmitter specificity is determined by postsynaptic target identity and extrinsic NTF signaling. (**C**). Summary data, mean peptidergic excitation of VF (number of action potentials induced) is unchanged by AChR antagonists, and influenced by NTFs. N ≥ 9. **P ≤ 0.002 (Independent Samples Kruskal–Wallis test). (**D**). Summary data, mean inhibition of RPeD1 (duration in s, reflects ACh + FMRF cotransmission or isolated FMRF transmission) is reduced by AChR antagonists in CM but not DM, indicating differential peptidergic inhibition influenced by NTFs. N ≥ 10. *P ≤ 0.010; ***P < 0.001 (Independent Samples Kruskal–Wallis test). (**E**). Summary data, mean amplitudes of ACh-PSPs are unchanged in CM or DM control. N ≥ 9. *P* = 0.490 (Independent Samples Kruskal–Wallis test). (**F**). Summary data, mean cholinergic excitation (number of action potentials induced), primarily biphasic cholinergic responses are observed in CM, and primarily inhibitory cholinergic responses are observed in DM. N ≥ 9. **P = 0.001; ***P < 0.001 (Independent Samples Kruskal–Wallis test). Error bars, SEM.

When cultured in CNS-conditioned media (CM), a NTF-rich media that recapitulates the in vivo environment, VD4-VF exhibited mixed cholinergic-peptidergic cotransmission, whereas most VD4-RPeD1 pairs exhibited primarily cholinergic transmission (Fig. 2A,C,D; Table S2A-B). As NTFs are well known to modulate synaptic function^40^, we next cultured pairs in NTF-deficient defined media (DM) to ask whether regulated cotransmission is solely target dependent, or whether it might also be influenced by extrinsic factors. Surprisingly, we observed a reversal of peptidergic phenotype, where most VD4-VF pairs exhibited primarily cholinergic transmission, and most VD4-RPeD1 pairs exhibited mixed cotransmission (Fig. 2B,C,D; Table S2A–B; see Fig. S2A-B). Analysis of the amplitudes of ACh-mediated postsynaptic potentials (PSPs) suggests that ACh release does not vary amongst VD4-VF and VD4-RPeD1 pairs cultured in CM or DM (Fig. 2E; Table S2B), while analysis of the postsynaptic ACh biphasic response indicates that excitatory, but not inhibitory, AChR expression is regulated by NTFs (Fig. 2F; Table S2B), in line with previous findings^27,35,41–43^. Taken together, these observations suggest that cholinergic and peptidergic transmission are tuned by distinct mechanisms.

**Figure 3:**
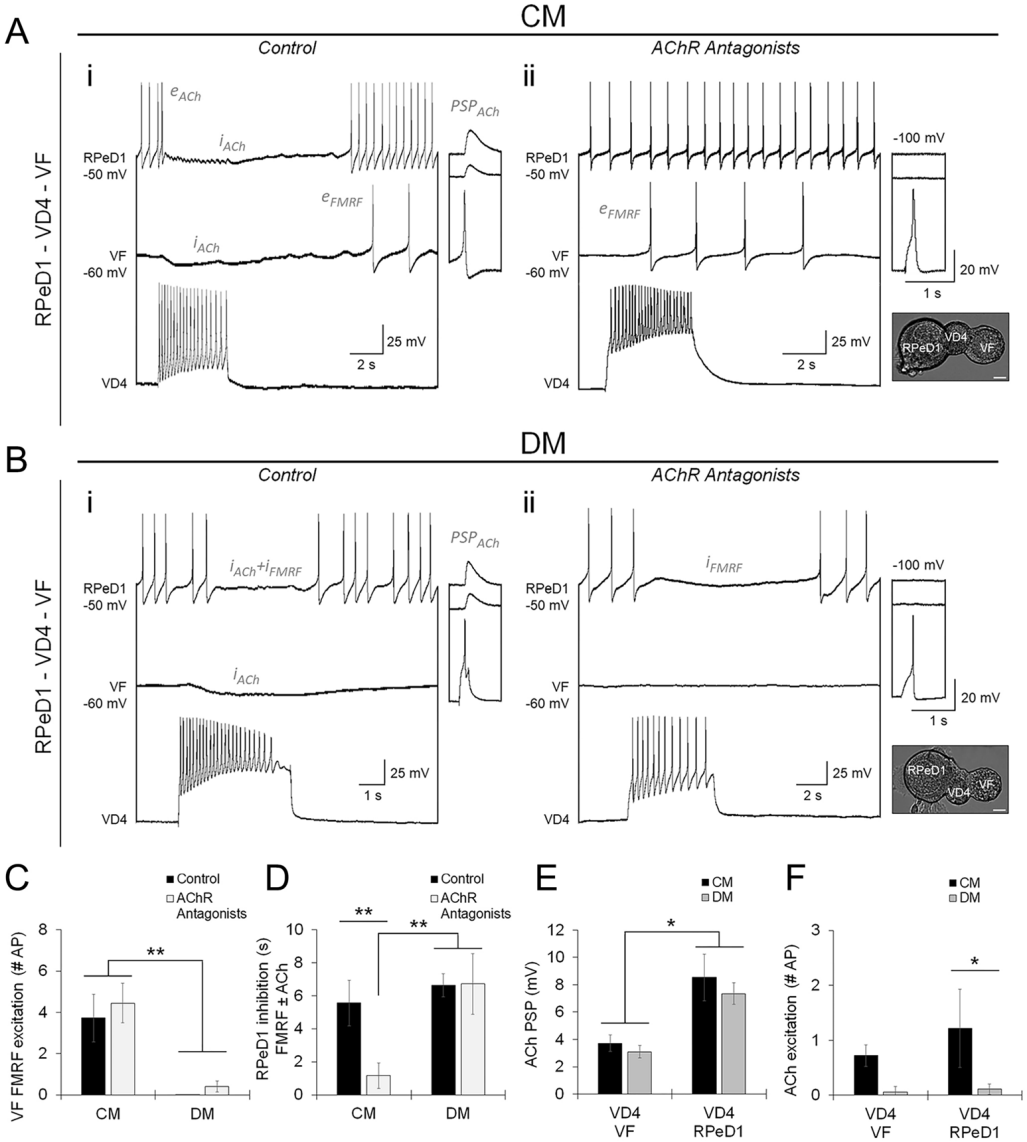
Target-specific use of classical and peptide cotransmitters is established by distinct mechanisms. (**A–B**). Characterization of cotransmission and isolated FMRF transmission by simultaneous intracellular current clamp recordings in neurons plated in a triple-soma configuration (image inserts; scale bar, 20 μm), performed in CM or DM control (**Ai** and **Bi**), or with AChR antagonists (**Aii** and **Bii**; 5 μM MLA, 10 μM TC, 20 μM TEA) to isolate peptidergic transmission. Inserts show ACh-PSPs (**i**; − 100 mV holding potential), inhibited by AChR antagonists (**ii**). Mixed cholinergic-peptidergic cotransmission is observed at VD4-VF synapses in CM, with inhibitory cholinergic and excitatory peptidergic responses (**Ai**,**ii**; i_ACh_, e_FMRF_), and at VD4-RPeD1 synapses in DM, with inhibitory cholinergic and peptidergic responses (**Bi**,**ii**; i_ACh_, i_FMRF_). Primarily cholinergic transmission is observed at VD4-RPeD1 synapses in CM (**Ai**,**ii**; e_ACh_, i_ACh_, absence of i_FMRF_), and VD4-VF synapses in DM (**Bi**,**ii**; i_ACh_, absence of e_FMRF_), (**C**). Summary data, mean peptidergic excitation of VF (number of action potentials induced) is unchanged by AChR antagonists, and influenced by NTFs. N ≥ 9. **P ≤ 0.002 (Independent Samples Kruskal–Wallis test). (**D**). Summary data, mean inhibition of RPeD1 (duration in s, reflects ACh + FMRF cotransmission or isolated FMRF transmission) is reduced by AChR antagonists in CM but not DM, indicating differential peptidergic inhibition influenced by NTFs. N ≥ 9. **P ≤ 0.009 (Independent Samples Kruskal–Wallis test). (**E**). Summary data, mean amplitudes of ACh-PSPs are unchanged in CM or DM control, but are larger at VD4-RPeD1 synapses than VD4-VF synapses, indicating that the use of classical and peptide cotransmitters in synaptic transmission are tuned by distinct synapse-specific mechanisms. N ≥ 9. *P ≤ 0.012 (Independent Samples Kruskal–Wallis test). (**F**). Summary data, mean cholinergic excitation (number of action potentials induced), primarily biphasic cholinergic responses are observed in CM, and primarily inhibitory cholinergic responses are observed in DM. N ≥ 9. *P = 0.023 (Independent Samples Kruskal–Wallis test). Error bars, SEM.

The next question was whether the specificity of classical and peptide cotransmission would be observed if VD4 innervated multiple postsynaptic targets in vitro. We therefore cultured VD4 simultaneously with VF and RPeD1 in a triple-soma configuration in CM, and found that the VD4-VF synapse exhibited mixed cotransmission, while the VD4-RPeD1 synapse exhibited primarily cholinergic transmission (Fig. 3A,C,D; Table S3A–B). Similarly in DM, the VD4-VF synapse exhibited primarily cholinergic transmission, while the VD4-RPeD1 synapse exhibited mixed cotransmission (Fig. 3B–D; Table S3A-B). In contrast to the single-synapse preparation, we found that the amplitude of ACh-mediated PSPs was smaller at VD4-VF than VD4-RPeD1 synapses in both CM and DM (Fig. 3E,F; Table S3B). This parallels the limited ACh transmission observed at VD4-VF synapses in situ (see Fig. 1 and Fig. S3), and suggests that ACh release in the context of synaptic competition is selectively modulated.

**Figure 4:**
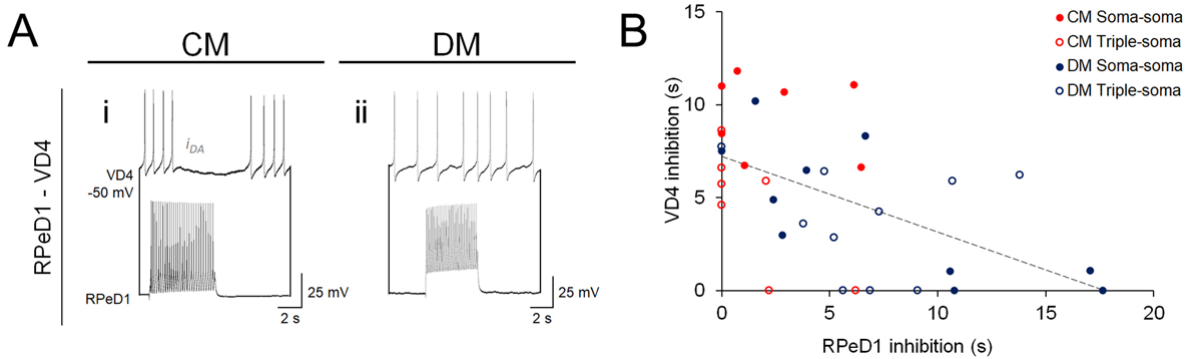
Reciprocal inhibitory transmission between VD4-RPeD1 is attenuated by FMRF neuropeptide transmission from VD4. (**A**). Simultaneous intracellular current clamp recordings in soma-soma paired VD4-RPeD1 neurons. Stimulation of RPeD1 induces DA-mediated inhibition in VD4 when pairs are cultured in CM (**i**; i_DA_), but not in DM (**ii**; absence of i_DA_), indicating that NTF-dependent regulation of FMRF peptidergic transmission (see also Figs. 2, 3) tunes the function of the reciprocal inhibitory synapse between VD4-RPeD1, which underlies respiratory central pattern generator (rCPG) activity and breathing behaviour in *Lymnaea* (see also Fig. 1). (**B**). Summary data, correlation of the mean dopaminergic synaptic response at RPeD1-VD4 synapses (y axis; VD4 inhibition) with the mean peptidergic synaptic response at VD4-RPeD1 synapses measured in the presence of AChR antagonists (x axis; RPeD1 inhibition; see also Figs. 2, 3). **P = 0.001; R = − 0.534; R^2^ = 0.2849 (Pearson correlation).

As FMRF neuropeptides have been shown to inhibit transmitter release^39^, we hypothesized that the degree of FMRF neuropeptide transmission would be inversely correlated with the degree of dopamine (DA) transmission at the reciprocal inhibitory synapse between VD4-RPeD1. Therefore, in parallel with monitoring ACh and FMRF neuropeptide transmission from VD4, we also evaluated DA transmission from RPeD1 in the above experiments. Most RPeD1-VD4 synapses exhibited strong inhibitory DA transmission when cultured in CM, whereas in DM most did not (Fig. 4A; Table S4). When we analyzed the duration of RPeD1 peptidergic inhibition vs the duration of VD4 dopaminergic inhibition, we found a significant negative correlation (Fig. 4B; Pearson correlation, R =  − 0.534, *P* = 0.001; R^2^ = 0.2849). This suggests that the context- and target-dependent regulation of FMRF neuropeptide transmission observed above may reflect differences in VD4′s FMRF neuropeptide release propensity, such that high-releasing synapses would occlude DA release from RPeD1 and low releasing synapses would facilitate DA release. If so, this NTF-dependent regulation of neuropeptide release holds important implications for the assembly, plasticity, and function of the *Lymnaea* rCPG network, and how the rhythmic network activity required for breathing behaviour is established and modulated^23,29^.

### Presynaptic inhibition of neuropeptide release defines cotransmitter specificity

**Figure 5:**
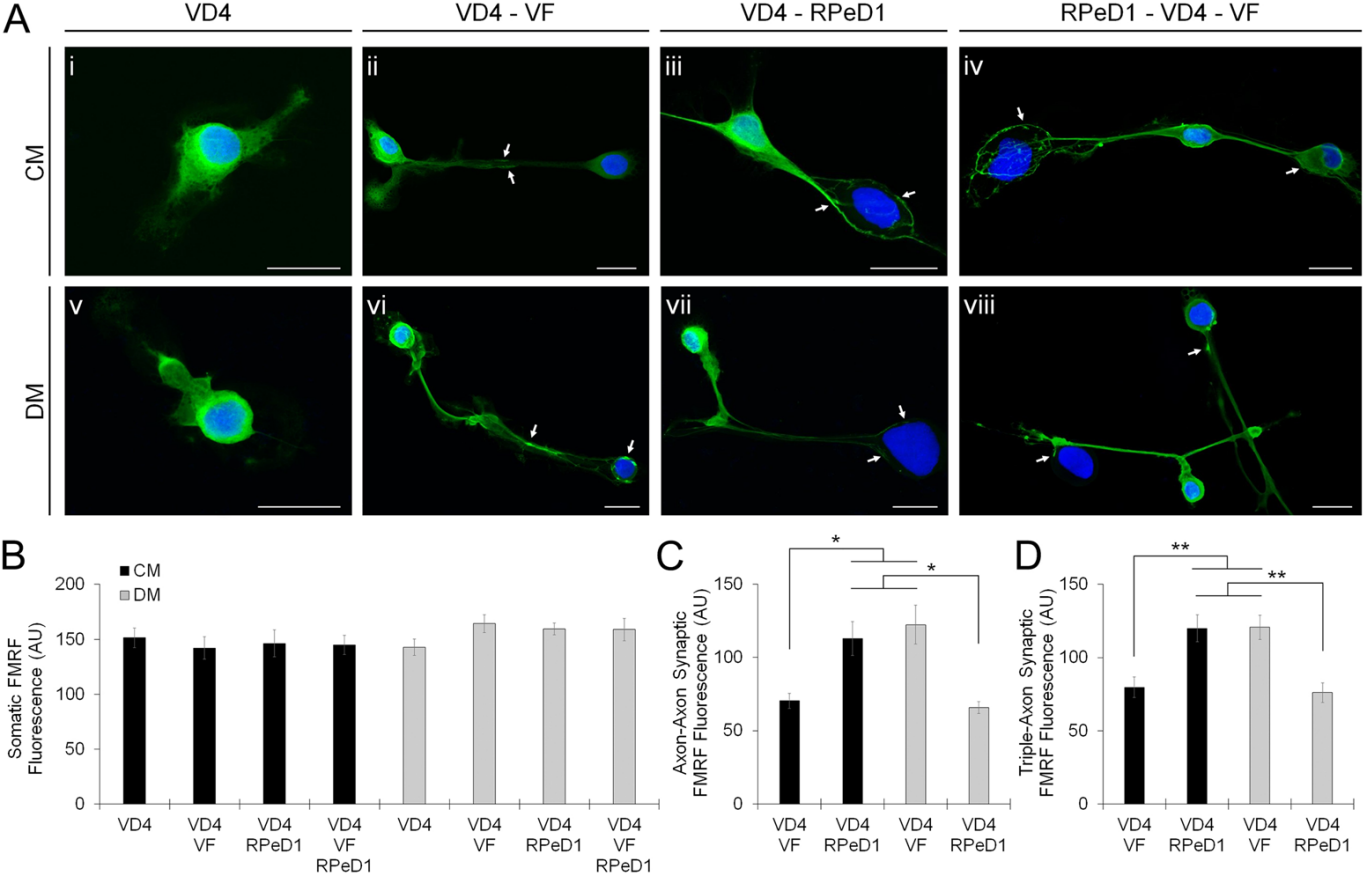
Presynaptic inhibition of neuropeptide release defines cotransmitter specificity. (**A**). ICC labeling of FMRF neuropeptides in VD4 neurons unpaired (**i**,**v**), VF-paired (**ii**,**vi**), RPeD1-paired (**iii**,**vii**), and dual RPeD1/VF-paired (**iv**,**viii**), cultured in CM (**i**–**iv**) or DM (**v**–**viii**). Images are merged with the nuclear stain DAPI. N ≥ 8. Scale bars, 50 μm. (**B**). Summary data, mean fluorescence intensity of somatic FMRF neuropeptides is uniform amongst various NTF and target cell contact conditions, indicating that translational FMRF neuropeptide expression in VD4 is unchanged (one-way ANOVA; *P* = 0.512). (**C**). Summary data, mean fluorescence intensity of synaptic FMRF neuropeptides (e.g. arrows) is increased in conditions that do not exhibit peptidergic transmission when VD4 is paired with a single postsynaptic target (VD4-VF DM, VD4-RPeD1 CM; see also Fig. 2). *P ≤ 0.031 (one-way ANOVA). (**D**). Summary data, mean fluorescence intensity of synaptic FMRF neuropeptides (e.g. arrows) is selectively increased at synapses that do not exhibit peptidergic transmission when VD4 is paired simultaneously with two postsynaptic targets (VD4-VF DM, VD4-RPeD1 CM; see also Fig. 3). **P ≤ 0.008 (one-way ANOVA), indicating that FMRF neuropeptides accumulate at presynaptic terminals with low neuropeptide release propensity. Error bars, SEM.

Considering the above observations, we postulated three scenarios to account for the apparent specificity of VD4′s FMRF neuropeptide release: (i) the translational expression of FMRF neuropeptides changes; (ii) FMRF neuropeptides are selectively targeted to specific presynaptic sites; or (iii) FMRF neuropeptides are selectively released from specific presynaptic sites. To explore these possibilities, we cultured VD4 neurons alone or with VF and/or RPeD1 postsynaptic targets in CM or DM, using an axon-axon configuration to facilitate visualization of synaptic sites. Using immunocytochemistry (ICC) and an antibody against the RF-NH_2_ moiety common to FMRF neuropeptides, we found a uniform intensity of somatic neuropeptide immunolabel amongst the culture conditions, suggesting that the translational expression of FMRF neuropeptides does not change (Fig. 5A,B; Table S5A). In paired neurons, conditions that exhibited limited peptidergic transmission (VD4-RPeD1 CM and VD4-VF DM) were characterized by a ‘hyper-innervation’ phenotype in which the intensity of the synaptic neuropeptide immunolabel was increased relative to the conditions that exhibited strong peptidergic transmission (VD4-VF CM and VD4-RPeD1 DM) (Fig. 5A,C,D; Table S5B). These results reveal that FMRF neuropeptides accumulate at presynaptic terminals with low release propensity, and that an inhibitory presynaptic mechanism regulates the target- and context-dependent specificity of peptidergic transmission.

### A Lymnaea synaptophysin homologue selectively inhibits neuropeptide release

We next sought to identify the molecular switch responsible for the selective facilitation and inhibition of peptidergic LDCV release. We focused on two synaptic vesicle protein (SVP)-dependent mechanisms that have been reported to selectively inhibit transmitter release. The first was the synaptotagmin (Syt) family of Ca^2+^ sensor SVPs, considering that different isoforms selectively facilitate or inhibit small synaptic vesicle (SSV) and LDCV release characteristics^44–46^. This has also been reported in the invertebrate literature^47–49^, indicating that the use of alternative Syt isoforms as selective regulators of transmitter release is an evolutionarily conserved strategy. The second was the vesicle integral membrane protein synaptophysin (Syp), which reversibly complexes with the v-SNARE synaptobrevin (Syb) when phosphorylated, rendering Syb unable to enter the fusogenic SNARE complex^50–52^.

**Figure 6:**
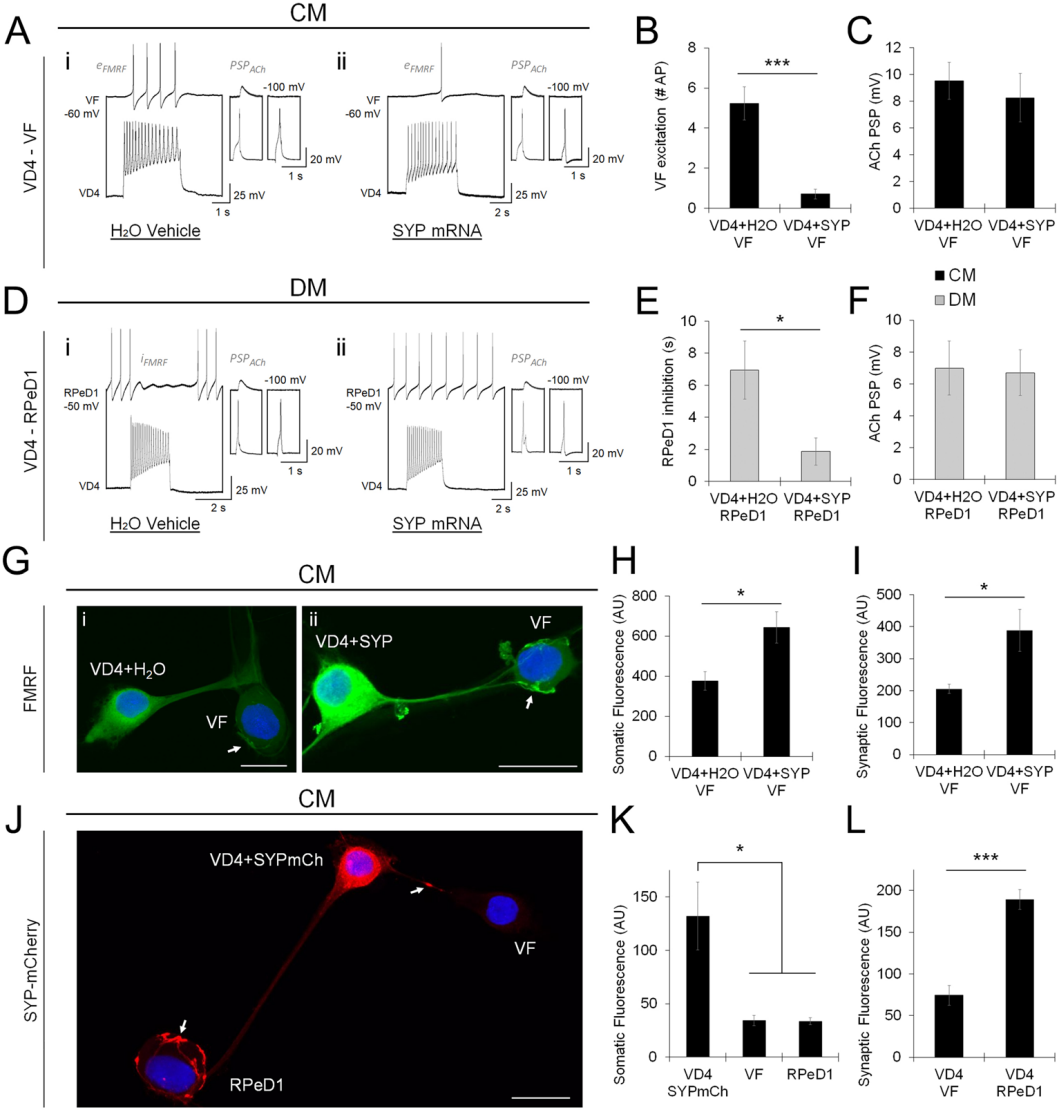
The *Lymnaea* synaptophysin homologue selectively inhibits neuropeptide release. (**A**). Simultaneous recordings of VD4-VF neurons paired in CM, made before and after the application of AChR antagonists (5 μM MLA, 10 μM TC, 20 μM TEA). VD4 was microinjected with H_2_O (**i**; vehicle control), or synthetic *L-*Syp mRNA (**ii**). Main panels show recordings made with AChR antagonists to isolate peptidergic transmission. Inserts show ACh-PSPs recorded in control conditions (left; -100 mV holding potential), which are inhibited by AChR antagonists (right). (**B**). Summary data, mean peptidergic excitation of VF (number of action potentials induced, AChR antagonist isolated), Syp overexpression reduces FMRF neuropeptide transmission. N ≥ 7. ***P < 0.001 (Mann–Whitney U test). (**C**). Summary data, mean ACh-PSP amplitudes of VD4-VF synapses, Syp overexpression does not affect ACh transmission. N ≥ 10. *P* = 0.613 (Independent Samples T-test). (**D**). VD4 neurons paired with RPeD1 in DM, as in (**A**). (**E**). Summary data, mean peptidergic inhibition of RPeD1 (duration in s, AChR antagonist isolated), Syp overexpression reduces FMRF neuropeptide transmission. N ≥ 9. *P = 0.025 (Mann–Whitney U test). (**F**). Summary data, mean ACh-PSP amplitudes of VD4-RPeD1 synapses, Syp overexpression does not affect ACh transmission. N ≥ 9. *P* = 0.898 (Independent Samples T-test). (**G**). ICC labeling of FMRF neuropeptides in VD4 neurons microinjected with H_2_O (**i**; vehicle control) or synthetic *L-*Syp mRNA (**ii**) and paired with VF in CM. Images are merged with the nuclear stain DAPI. Scale bars, 50 μm. (**H**). Summary data, mean fluorescence intensity of somatic FMRF neuropeptides is increased by Syp overexpression. N ≥ 6. *P = 0.017 (Independent Samples T-test). (**I**). Summary data, mean fluorescence intensity of synaptic FMRF neuropeptides is increased by Syp overexpression. N ≥ 6. *P = 0.028 (Independent Samples T-test). (**J**). ICC labeling of VD4 neurons microinjected with synthetic *L-*Syp-mCherry mRNA and paired with VF and RPeD1 in CM. Scale bar, 50 μm. (**K**). Summary data, mean fluorescence intensity of somatic Syp-mCherry is higher in microinjected VD4 than non-injected VF and RPeD1. N = 3. *P ≤ 0.023 (one-way ANOVA). (**L**). Summary data, mean fluorescence intensity of synaptic Syp-mCherry is selectively enhanced at synapses with RPeD1, which do not exhibit peptidergic transmission and show accumulation of FMRF neuropeptides (see also Figs. 2, 3, 5). ***P < 0.001 (Independent Samples T-test). Error bars, SEM.

To determine the influence of the *Lymnaea* homologues of Syt and Syp on cotransmitter release, we first identified and cloned them from a *Lymnaea* CNS cRNA library (see Fig. S6A–C), then microinjected synthetic mRNA into VD4 to evaluate the effects of their overexpression on classical and peptidergic transmission. VD4-VF pairs were cultured in CM and VD4-RPeD1 pairs were cultured in DM to facilitate formation of synapses with high neuropeptide release propensity. In both cases, overexpression of Syp reduced FMRF neuropeptide transmission relative to H_2_O vehicle control (Fig. 6A,B,D,E; Table S6A), but had no effect on the amplitude of ACh-mediated PSPs (Fig. 6C,F; Table S6B), whereas Syt isoforms affected ACh, but not FMRF neuropeptide transmission (see Fig. S6D). Using ICC in axon-axon pairs, we found that Syp overexpression increased the intensity of the somatic and synaptic FMRF neuropeptide immunolabel relative to H_2_O vehicle control (Fig. 6G–I; Table S6C), while the Syt isoforms had no effect (see Fig. S6E). These data suggest that Syp acts as an inhibitory regulator of LDCVs, which tunes synaptic cotransmission by selectively attenuating neuropeptide release.

Next, if Syp mediates target-specific inhibition of FMRF neuropeptide release, we reasoned it would sort to synapses in a manner resembling the distribution patterns observed for FMRF neuropeptides. To characterize Syp localization, we generated a C-terminal mCherry tagged construct. VD4 was microinjected with synthetic Syp-mCherry mRNA and paired in a dual synapse axon-axon configuration with VF and RPeD1 in CM. We performed ICC using an α-mCherry antibody, and found that Syp synaptic fluorescence intensity was higher at synapses with RPeD1 than with VF (Fig. 6J–L; Table S6D), mimicking the selective accumulation of FMRF neuropeptides observed at low release propensity synapses (see Fig. 5).

Taken together, these observations support a Syp-dependent mechanism for the inhibitory regulation of neuropeptide release, and suggest that presynaptic peptide cotransmitter specificity requires synapse-specific populations of release-competent and -incompetent LDCVs.

### NTF and target-specific regulation of GSK-3 activity determine neuropeptide release competency

We next sought to identify the molecular signaling cascades that define FMRF neuropeptide release characteristics, reasoning that it likely involves synapse-delimited post-translational modification (PTM) of Syp. GSK-3 has been shown to promote Syp-Syb complexing via phosphorylation of Syp^53^, and GSK-3 activity is known to be high under basal conditions and inhibited in response to receptor tyrosine kinase (RTK) activation, which transduce NTF signaling^54,55^. This implicates a model for context-dependent inhibitory regulation of neuropeptide release in which (i) GSK-3 activity is high in the absence of NTFs, causing phosphorylation of Syp and enhancing formation of the Syp-Syb complexes that inhibit LDCV fusion, and (ii) GSK-3 activity is low in the presence of NTFs, reducing Syp phosphorylation and promoting Syp-Syb dissociation that promotes neuropeptide release.

**Figure 7:**
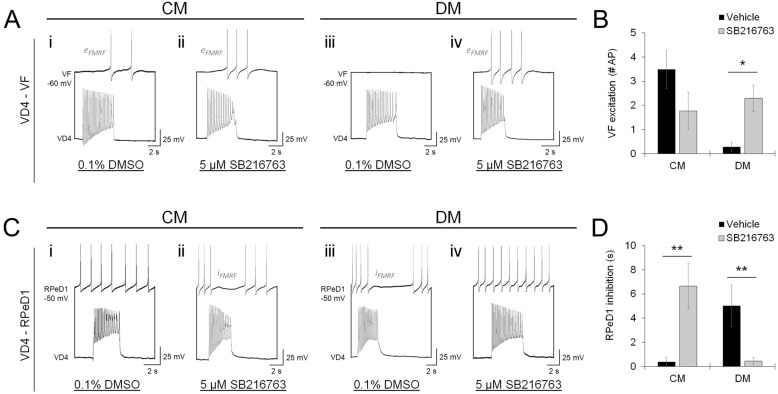
GSK-3 activity defines neurotrophic factor-dependent regulation of presynaptic neuropeptide release competency. (**A**). Recordings of VD4-VF soma-soma paired neurons cultured in CM (**i,ii**) or DM (**iii,iv**), in the presence of 0.1% DMSO (**i,iii**; vehicle control) or 5 μM SB 216763 (**ii,iv**; GSK-3 inhibitor). Recordings were made in the presence of AChR antagonists (5 μM MLA, 10 μM TC, 20 μM TEA) to isolate peptidergic transmission. In CM, where GSK-3 is inhibited by NTF/RTK signaling, SB 216763 has no effect on the incidence of peptidergic transmission. In DM, where basal GSK-3 activity is high, inhibition by SB 216763 induces neuropeptide release. This indicates that neuropeptide release at VD4-VF synapses is defined by the effect of NTF/RTK signaling on GSK-3 activity. (**B**). Summary data, mean peptidergic synaptic response in VF measured in the presence of AChR antagonists. N ≥ 10. *P = 0.013 (Independent Samples Kruskal–Wallis test). (**C**). Recordings of peptidergic transmission between VD4-RPeD1 neurons, as in (**A**). In CM, GSK-3 inhibition by SB 216763 induces peptidergic transmission, whereas it is inhibited in DM. This indicates that a molecular signaling interaction between VD4-RPeD1 induces an atypical regulation of GSK-3 activity and neuropeptide release. (**D**). Summary data, mean peptidergic synaptic response in RPeD1, as in (**B**). N ≥ 8. **P ≤ 0.009 (Independent Samples Kruskal–Wallis test). Error bars, SEM.

To test this experimentally, we cultured VD4-VF and VD4-RPeD1 pairs in CM or DM, in the presence of a GSK-3 inhibitor (5 μM SB 216763) or vehicle control (0.1% DMSO). When cultured in CM, VD4-VF peptidergic transmission was not impacted by GSK-3 inhibition, while in DM, GSK-3 inhibition induced peptidergic transmission (Fig. 7A,B; Table S7A). This suggests that neuropeptide release characteristics at VD4-VF synapses are defined by NTF-dependent regulation of GSK-3 activity. In VD4-RPeD1 pairs, however, we found that GSK-3 inhibitors reversed neuropeptide release characteristics in both CM and DM (Fig. 7C,D; Table S7B). This suggests that GSK-3 activity at VD4-RPeD1 synapses is not regulated by the conventional NTF/RTK pathway, and implicates a target-dependent molecular signaling interaction that acts to define neuropeptide release competency.

### Target-specific retrograde arachidonic acid signaling defines neuropeptide cotransmitter release

**Figure 8:**
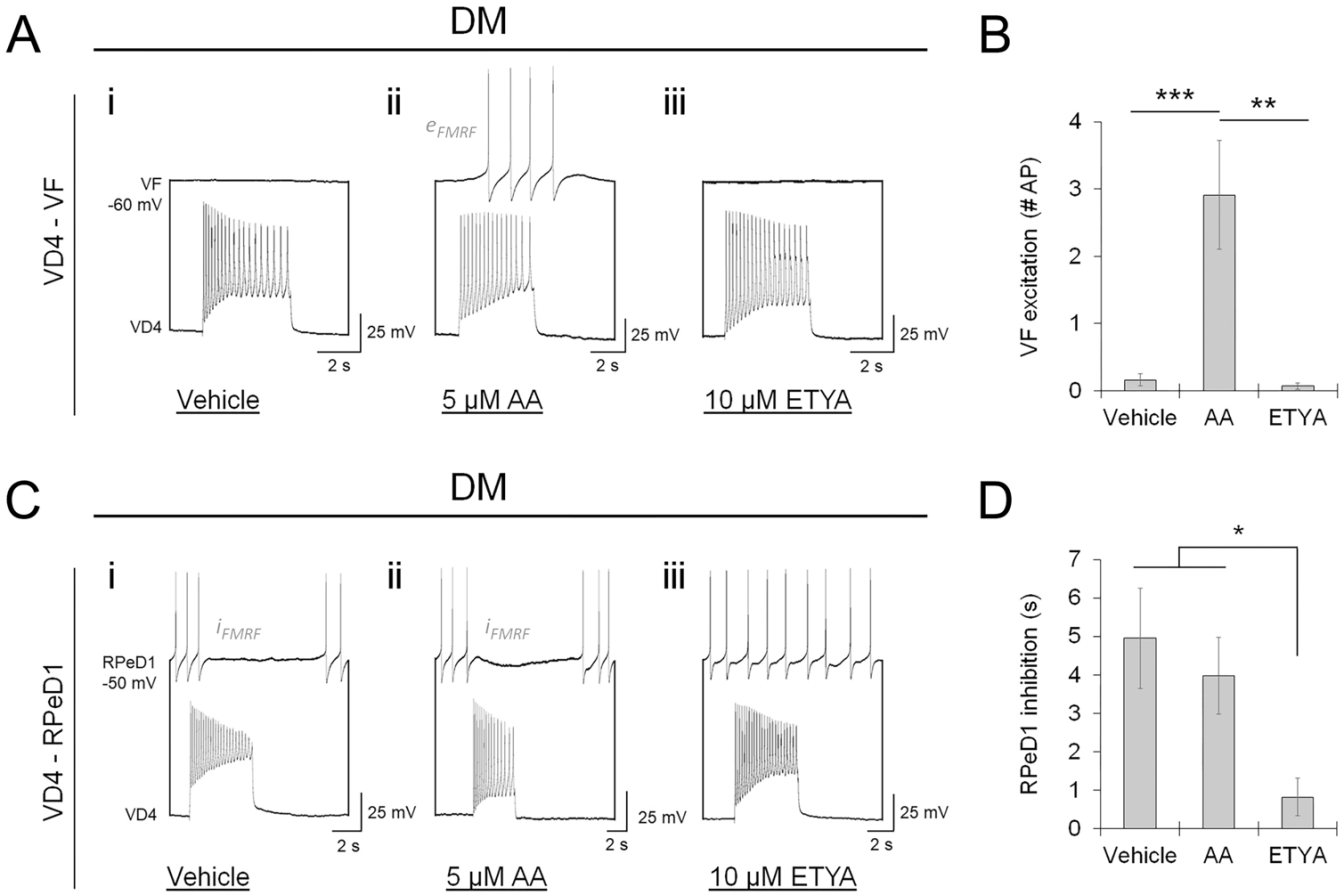
Retrograde arachidonic acid signaling defines target-dependent regulation of presynaptic neuropeptide release competency. (**A**). Recordings of VD4-VF soma-soma paired neurons cultured in DM + vehicle control (**i**; 0.1% DMSO + 0.1% Tocrisolve), DM + 5 μM Arachidonic Acid (**ii**; free AA), or DM + 10 μM ETYA (**iii**; inhibitor of AA metabolism). Recordings were made in the presence of AChR antagonists (5 μM MLA, 10 μM TC, 20 μM TEA) to isolate peptidergic transmission. AA exposure induces the release of neuropeptides, whereas the inhibitor ETYA has no effect, indicating that VD4-VF pairs do not exhibit retrograde AA signaling interactions. (**B**). Summary data, mean peptidergic synaptic response in VF measured in the presence of AChR antagonists. N ≥ 13. **P = 0.001; ***P < 0.001 (Independent Samples Kruskal–Wallis test). (**C**). Recordings of peptidergic transmission between VD4-RPeD1 neurons, as in (**A**). AA exposure has no effect, whereas the inhibitor ETYA inhibits neuropeptide release, indicating that a target-specific retrograde AA signal from RPeD1 regulates neuropeptide release from VD4. (**D**). Summary data, mean peptidergic synaptic response in RPeD1, as in (**B**). N ≥ 15. *P = 0.010 (Independent Samples Kruskal–Wallis test). Error bars, SEM.

The release of FMRF neuropeptides from VD4 produces excitation in VF and inhibition in RPeD1, and the metabotropic receptors are coupled to distinct G proteins in these two postsynaptic targets (see Fig. S2B). As GPCRs activate a range of metabolic signaling cascades that act locally and trans-synaptically, we reasoned that cotransmitter specificity was elicited by differences in FMRF transmitter-receptor interactions and cell–cell signaling. Retrograde signaling by membrane-permeable second messengers is well known to influence synaptic function^56^. In particular, the generation of arachidonic acid (AA) metabolites has been linked to the actions of FMRF neuropeptides^57,58^, and was previously implicated in the function of reciprocal VD4-RPeD1 synapses^59^. Since we observed an atypical GSK-3 signature at VD4-RPeD1 synapses, we wondered whether target-specific retrograde AA might influence neuropeptide release. Therefore, VD4-VF and VD4-RPeD1 pairs were cultured in DM, to avoid NTF/RTK signaling, and supplemented with free arachidonic acid (AA, 5 μM), ETYA (10 μM, an inhibitor of AA metabolism), or vehicle control (0.1% DMSO + 0.1% Tocrisolve 100). Relative to control, AA induced peptidergic transmission at VD4-VF pairs, while the inhibitor ETYA had no effect (Fig. 8A,B; Table S8A), indicating that AA metabolism is not endogenous to FMRF neuropeptide signaling between VD4-VF. By contrast, AA supplementation did not influence peptidergic transmission in VD4-RPeD1 pairs, whereas ETYA inhibited peptidergic transmission (Fig. 8C,D; Table S8B), indicating that FMRF neuropeptide signaling between VD4-RPeD1 induces AA metabolism.

**Figure 9:**
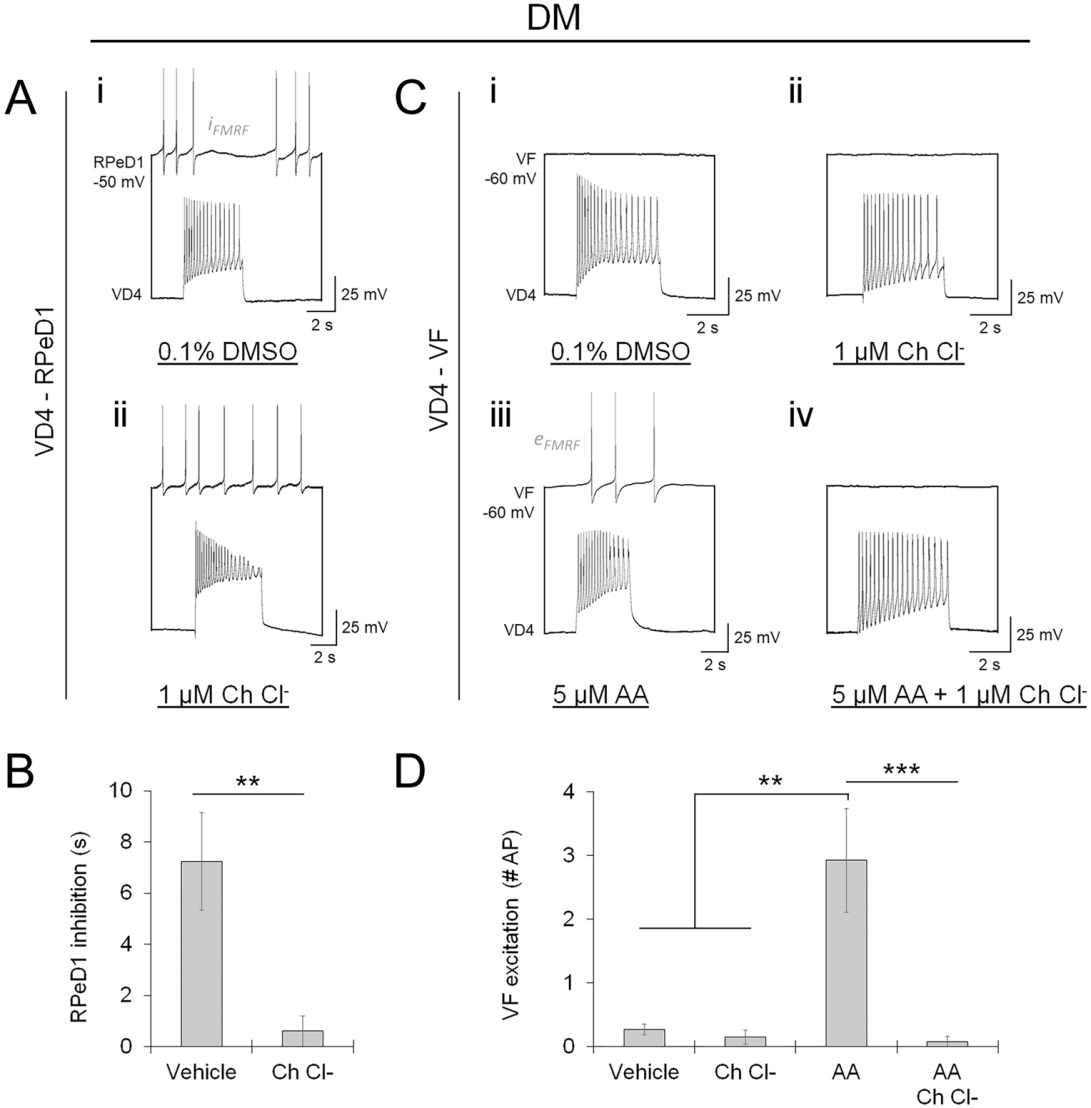
A PKC family kinase mediates arachidonic acid-dependent regulation of presynaptic neuropeptide release competency. (**A**). Recordings of VD4-RPeD1 soma-soma paired neurons cultured in DM + vehicle control (**i**; 0.1% DMSO), or DM + 1 μM Chelerythrine Chloride (**ii**; Ch Cl^−^, PKC inhibitor). Recordings were made in the presence of AChR antagonists (5 μM MLA, 10 μM TC, 20 μM TEA) to isolate peptidergic transmission. Antagonism of PKC inhibits neuropeptide release, indicating that it perturbs retrograde AA signaling interactions between VD4-RPeD1. (**B**). Summary data, mean peptidergic synaptic response in RPeD1 measured in the presence of AChR antagonists. N ≥ 8. **P = 0.007 (Mann–Whitney U test). (**C**). Recordings of peptidergic transmission between VD4-VF neurons, as in (**A**). VD4-VF neurons were cultured in DM + vehicle control (**i**; 0.1% DMSO + 0.1% Tocrisolve; data in part is repeated here from Fig. 8A), DM + 1 μM Ch Cl^−^ (**ii**), DM + 5 μM AA (**iii**; data is repeated here from Fig. 8A), or DM + 5 μM AA + 1 μM Ch Cl^−^ (**iv**). Antagonism of PKC inhibits the induction of neuropeptide release by AA, but otherwise has no effect on peptidergic transmission between VD4-VF. (**D**). Summary data, mean peptidergic synaptic response in VF, as in (B). N ≥ 10. **P ≤ 0.002, ***P < 0.001 (Independent Samples Kruskal–Wallis test). Error bars, SEM.

Since AA has been shown to activate protein kinase C (PKC) family kinases^60^, which have been reported to influence GSK-3 activation^61,62^, we suspected that target-specific retrograde AA signaling influenced peptidergic transmission via PKC. VD4-VF and VD4-RPeD1 pairs were cultured in DM in the presence of the inhibitor Chelerythrine chloride (Ch Cl^−^; 1 μM) to determine whether PKC is a molecular target for AA. Relative to vehicle control (0.1% DMSO), PKC inhibition prevented peptidergic transmission at VD4-RPeD1 synapses (Fig. 9A,B; Table S9A). We found no effect of PKC inhibition on peptidergic transmission at VD4-VF synapses; however, it negated the induction of peptide release via AA (Fig. 9C,D; Table S9B).

**Figure 10:**
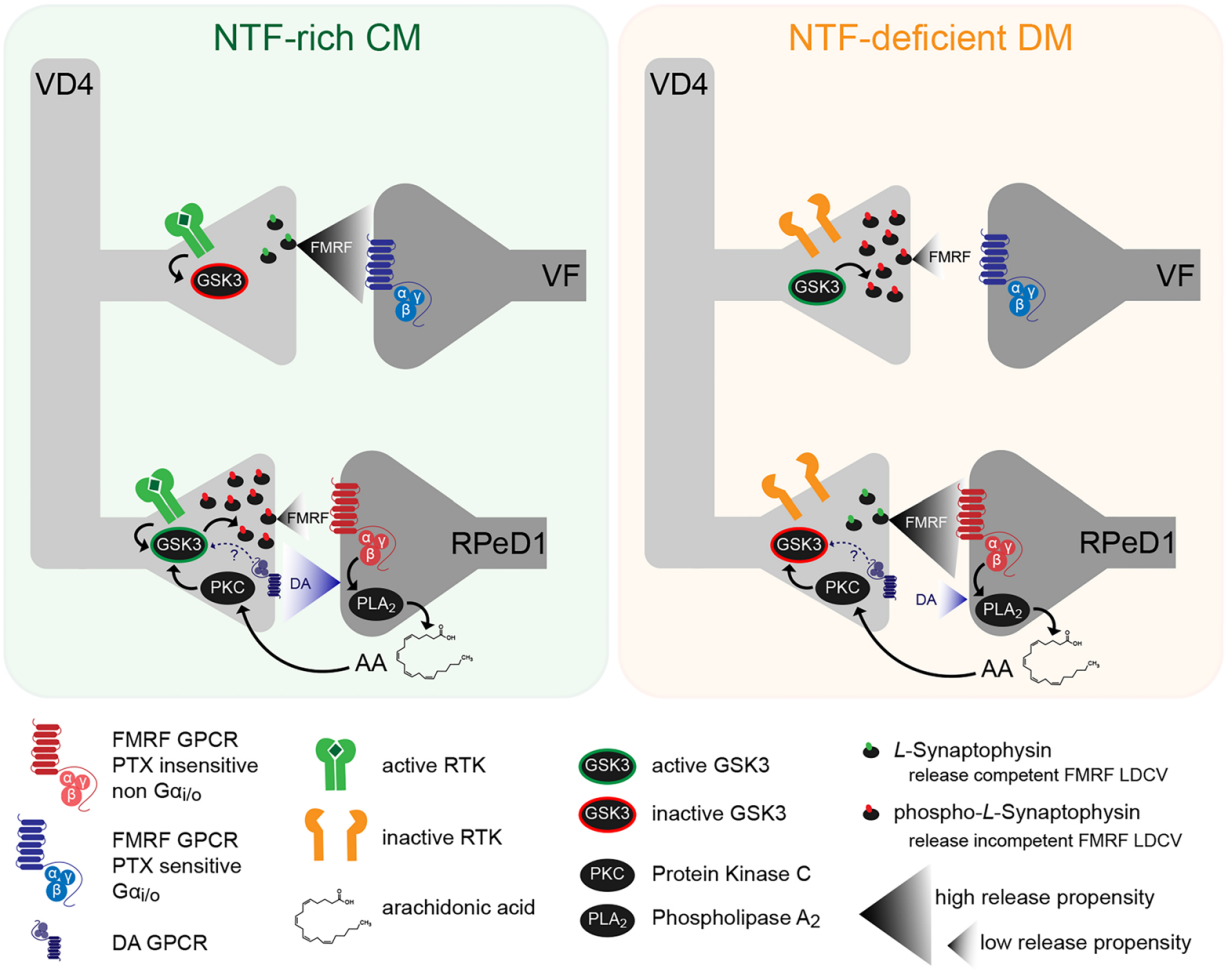
Proposed model for the molecular signaling network underlying presynaptic neuropeptide cotransmitter specificity.

These data reveal that target-specific retrograde AA signaling acts via a PKC family kinase to define the cotransmitter release properties of innervating presynaptic terminals. Taken together, our observations on the regulation of peptide cotransmitter use at individual synapses uncover a previously undefined molecular signaling network that tunes the cotransmitter release characteristics of individual presynaptic terminals (Fig. 10).

## Discussion

In this study we sought to identify the cellular and molecular mechanisms that specify the differential use of neurotransmitters at the individual presynaptic terminals formed by a cotransmitting neuron—a key mechanism for synaptic heterogeneity that underlies the selective processing and relay of information within neuronal circuits. Here, using *Lymnaea* cardiorespiratory neurons, we have identified (i) a new component of NTF-dependent plasticity that selectively defines peptidergic cotransmission, (ii) a novel function for the *Lymnaea* synaptophysin homologue in the inhibitory regulation of neuropeptide release, and (iii) a role for AA metabolites as a target-specific retrograde signaling mechanism that establishes cotransmitter specificity. While we do not exclude the possibility that there may be concurrent changes in the expression or function of postsynaptic receptors, our observations of presynaptic neuropeptide accumulation and the inhibitory role of synaptophysin identify the presynaptic terminal as an important locus for target- and context-dependent regulation of cotransmission. Taken together, our findings define a molecular signaling network that underlies presynaptic cotransmitter specificity, and can allow for the context-dependent tuning of synaptic network function underlying the plasticity of patterned motor behaviours. Through this pathway, the release characteristics of individual synapses can be tuned to meet the dynamic functional requirements of neuronal networks set by an animal’s demand for behavioural plasticity.

### Peptidergic cotransmission

One particular question in the field of cotransmission that has remained unresolved is whether the synaptic colocalization of multiple neurotransmitter substances necessarily implies their corelease^63^. Here, we show that peptide cotransmitter release at inter-neuronal synapses is regulated by context-dependent inhibition. FMRF neuropeptides accumulated at functionally non-peptidergic presynapses, while functionally peptidergic presynapses showed comparatively little localization, indicating that colocalization does not imply cotransmission^63^. Consistent with this notion, a previous study on the *Drosophila* neuromuscular junction showed that neuropeptide stores are low in resting terminals, where they are supplied in a use-dependent manner by the activity-dependent capture of rapidly transiting LDCVs^64^. This suggests that, in contrast to classical transmission, peptidergic transmission may be sufficiently maintained in the absence of a sizeable reserve of LDCVs in the presynaptic bouton.

The main focus of our study was to define the molecular mechanisms that selectively regulate neuropeptide cotransmitter release, however, our observation that the release of ACh from VD4 is tuned independently from FMRF neuropeptide release warrants further investigation to advance our understanding of the dynamic regulation of cotransmitter release and presynaptic heterogeneity. We suspect that there are a number of mechanisms, likely involving target-specific signaling interactions and cross-talk amongst signaling networks, at play. First, work in *Drosophila* has demonstrated that neuropeptide autoreceptor signaling via presynaptic convergence adjusts cotransmitter output by enhancing the release of classical transmitters to accelerate the circadian rhythm directing sleep–wake behaviour^65^. Second, we have previously reported that ACh release competency in VD4 is scaled by presynaptic cAMP/PKA activation during competition for postsynaptic innervation^66^. Third, in the murine hippocampus, target-specific retrograde AA signaling has been found to induce presynaptic spike broadening by local inhibition of voltage-gated potassium channels, leading to synaptic facilitation^67^. Taken together, these observations indicate that a variety of mechanisms differentiate the function of individual presynaptic terminals to establish synaptic heterogeneity and enable selective information processing in neuronal circuits.

### Synapse-specific inhibition of neuropeptide release by synaptophysin

Considering our observations on role of the *Lymnaea* Syp homologue in inhibiting LDCV release, it is important to note that mammalian Syp has been widely regarded to be specifically localized to SSVs, and absent from LDCVs^68^. If the mechanism described here is conserved, one possibility is that inhibitory regulation of LDCVs in mammals is mediated by another member of the Syp protein family. Another possibility raised by studies in neuroendocrine cells is that Syp is present at varying concentrations in LDCVs, and at lower concentrations than in SSVs^69,70^. This is consistent with a study on the protein composition of single SVs reporting that some SVPs, including Syt I, are sorted with high precision, whereas others, including Syp and Syb, show significant intervesicle variability^71^. In view of our findings on the inhibitory regulation of neuropeptide release by *Lymnaea* Syp, it seems that synaptic cotransmitter specificity might be achieved by two complementary mechanisms: the differential trafficking of release competent (Syp-) or release incompetent (Syp +) LDCVs to presynaptic terminals; and synapse-delimited PTM of Syp that alters the release competency of Syp + LDCVs.

Regarding the differential trafficking of Syp + and Syp− LDCVs, N-glycosylation, a PTM that occurs in the endoplasmic reticulum and affects protein sorting and trafficking, has been shown to be essential for targeting Syp, but not other SVPs such as Syt, out of the cell body and to the synapse^72^. This suggests that N-glycosylation might act as a selectivity filter to govern the amount of Syp incorporated during LDCV biogenesis, which could regulate the release competency of LDCVs and also account for the controversial and variable presence of Syp on LDCVs. Downstream of biogenic sorting, the synapse-specific targeting of cargo, including LDCVs, is a second highly regulated process that differentiates the composition and function of individual presynaptic terminals during synaptogenesis and synaptic plasticity^64,73,74^. Considering our findings, it may be that NTF/RTK and retrograde AA signaling establish synapse-specific populations of release-competent and -incompetent LDCVs by influencing Syp N-glycosylation and the selective trafficking or capture of Syp + vs Syp- LDCVs at presynaptic terminals.

Regarding synapse-delimited PTM of Syp, Syp-Syb affinity has been reported to be modulated by GSK-3 activity, where phosphorylation of Syp by GSK-3 inhibits Syb dissociation and attenuates transmitter release^53^. Here, we found that target-dependent regulation of neuropeptide release involved the regulation of GSK-3 activity by NTF/RTK signaling, and PKC family kinase activity by retrograde AA signaling. The inhibition of GSK-3 by RTKs is known to involve the Par3-Par6-aPKC cell polarity complex^61^. During CNS development, the asymmetric accumulation of these polarity proteins mediates neurogenic divisions by neural progenitors, and regulates microtubule dynamics underlying axogenesis and directed outgrowth^61^. An attractive mechanism for the local regulation of neuropeptide release by GSK-3-dependent phosphorylation of Syp would be the differential localization of the Par3-Par6-aPKC cell polarity complex underlying the functional heterogeneity of individual presynaptic terminals.

### Functional implications for context-dependent tuning of peptidergic transmission

In *Lymnaea*, NTF-dependent synaptic network plasticity has been proposed to mediate the seasonal regulation of heart rate and breathing behaviour during aestivation^75^. The regulation of FMRF neuropeptide release described here identifies a new mechanism with significant potential to underlie the plasticity of cardiorespiratory behaviour induced by NTFs. Increases or decreases in NTF/RTK signaling would differentially affect FMRF neuropeptide release at VD4′s excitatory synapse with VF to promote or inhibit cardiac drive, and the reciprocal inhibitory synapse with RPeD1 to facilitate or attenuate rCPG rhythmogenesis and breathing behaviour.

In the stomatogastric ganglion (STG) of the crab *Cancer borealis*, a cotransmitting projection neuron (MPN) selectively activates and inhibits the expression of pyloric and gastric mill motor patterns using its neuropeptide cotransmitter at synapses within the STG network and its small molecule cotransmitter at synapses with projection neurons in the commissural ganglia^6^. Thus, it seems that the selective use of neuropeptide and classical small molecule cotransmitters with distinct postsynaptic targets is a common mechanism underlying the expression of patterned motor behaviour in invertebrates. It would next be pertinent to determine whether an analogous mechanism involving target-specific retrograde AA signaling and Syp-dependent inhibition of LDCV fusion may be involved in expressing gastric mill motor patterns in the *Cancer* STG, and whether this may be a more general mechanism for the target-specific regulation of cotransmitter release underlying the appropriate expression and context-dependent plasticity of patterned motor behaviours across species.

The regulated complexing interactions of Syp and Syb have been suggested to act in establishing a reserve pool of SVs that can be recruited by synaptic activity^76^. Applied to the regulated secretion of neuropeptides, this adjustable reserve function would have interesting implications for our understanding of the dynamic propensity of neurons to modulate the release of their neuropeptides. In *Lymnaea*, release of the ovulation hormone requires priming by a prolonged period of coordinated network spiking activity^77^. Activity-dependent dephosphorylation of Syp might switch release-incompetent LDCVs to release-competent ones, perhaps involving activity-dependent NTF/RTK signaling. This mechanism would allow resting secretory terminals to accumulate neurohormones to levels sufficient for sustaining endocrine signaling, and maintain the system in an energetically favorable ‘primed-but-off’ state that would decouple neurohormone release from slower somal-dependent processes.

In mice Syp knockout does not result in any readily apparent structural or synaptic deficits, however, changes in short- and long-term plasticity and hippocampus-dependent behaviour have been reported^78–80^. These findings suggest that mammalian Syp is an essential regulator of synaptic plasticity, although previous studies have been unable to elucidate the underlying mechanism. Moreover, Syp is one of the genetic mutations implicated in human X-linked intellectual disability^81^. A compelling possibility raised by our work is that the synaptic plasticity deficits linked to mammalian Syp family proteins may involve the aberrant release of peptide cotransmitters. Further characterization of this Syp-dependent mechanism for regulated neuropeptide release, as it pertains to synaptic specificity and plasticity, is therefore likely to offer new fundamental insights into neurological disorders.

## Methods

### Animals and neuronal cell culture

*Lymnaea stagnalis* were raised under standard conditions in freshwater aquaria at room temperature (~ 22 °C) on a diet of lettuce. Animals (6–8 weeks old) were anesthetized with a 10% Listerine solution (10 min) and sacrificed by dissection of the CNS in normal *Lymnaea* saline. Identified neurons VD4, VF and RPeD1 were isolated from trypsinized CNS by suction applied through a glass pipette and then cultured on poly-L-lysine coated glass culture dishes, as previously described in detail^23,82^. Neurons were maintained overnight (15–20 h) in defined media (DM, NTF-deficient media; L-15; Life technologies; special order) or NTF-rich CNS conditioned media (CM, CNS-incubated DM), as described elsewhere^41^.

### Molecular biology

The *Lymnaea* Syp homologue was identified from the *Lymnaea* EST library^83^ using a BLAST search (blastn, NCBI) against mouse Syp (Accession number NM_009305), which revealed a single hit containing a complete protein-coding sequence (Accession number ES572211). We screened clones of the *Lymnaea* Syt I homologue (Accession number AF484090)^84^, and identified C2B-α (Syt-α) and C2B-β (Syt-β) isoform splice variants, each with a frequency of ~ 50%, suggesting that both transcripts are abundant in the CNS. The coding sequences for Syp, Syt-α, and Syt-β were cloned from *Lymnaea* CNS cDNA using Platinum *Taq* DNA polymerase (Invitrogen), and then subcloned into pBlueScript SK- (Clontech), as described elsewhere^30^. A C-terminal mCherry tag was introduced to Syp with site overlap extension (SOE) PCR using the KAPA HiFi HotStart ReadyMix PCR kit (Kapa Biosystems), as described previously^35^. See Supplementary Experimental Procedures for primer sequences (Table S10).

Synthetic mRNA was made with the mMESSAGE mMACHINE T7 Ultra transcription kit, according to manufacturer’s instructions (Ambion). Purified synthetic mRNA was dissolved in molecular-grade water (3–6 μg/μL) and microinjected into VD4 neurons using a sterile low resistance glass electrode (10 pulses, 250 ms, 10 PSI). Molecular-grade water was used as a vehicle control. Microinjections were performed shortly after neurons were plated, allowing time for the cells to adhere to the poly-L-lysine coated coverslip (~ 2 h). Synapses developed overnight in CM or DM.

### Immunocytochemistry and imaging

Neurons were fixed for 30 min with 1% paraformaldehyde and 0.2% picric acid in 1xPBS, then permeabilized for 1 h with incubation media (IM) containing 0.2% Triton, 5% fetal calf serum, and 0.25% fish gelatin in 1xPBS. Primary antibodies against FMRF neuropeptides (detects the common Arg-Phe-NH_2_ moiety^85^), or mCherry (BioVision, 5993) were used at 1:500 in IM for 1 h. The secondary antibody (Invitrogen, Alexa Fluor 546 Goat α Rabbit) was used at 1:100 in IM for 1 h. Three 15 min washes in 1xPBS were performed between each incubation, and all incubations were performed at room temperature. Cells were mounted with MOWIOL containing DAPI.

Samples were imaged with an A1R MP microscope using a CFI Plan Fluor 20x/0.75 MI objective (Nikon). Laser excitation wavelengths were 402 and 561, in series, and emission wavelengths were collected through 450/50 and 595/50 filter cubes (Nikon). For quantitative analysis, imaging parameters were kept the same amongst relevant samples. Images were acquired with NIS Elements v4.13.00 (Nikon), and intensity analysis was performed with ImageJ (NIH). To determine the synaptic localization of FMRF neuropeptides and Syp-mCherry, we measured the mean fluorescence intensity at 3 presynaptic varicosities per pair. Presynapses were defined by their distinct morphology coincident with FMRF or mCherry immunoreactivity. We have previously combined this approach with FM1-43 dye uptake to verify that morphologically defined varicosities are functional presynaptic terminals^35^. A small number of samples in which presynaptic varicosities were not apparent were excluded from analyses.

### Electrophysiology

Simultaneous intracellular current clamp recordings and Lucifer yellow (LY) dye injections were performed as previously described for in vitro^41^ and in situ^86^ preparations. Briefly, for cultured neurons, cells were maintained in CM or DM (in mM: 40 NaCl; 1.7 KCl; 4.1 CaCl_2_; 1.5 MgCl_2_; 5 HEPES; pH 7.9), and glass microelectrodes (resistance 20–50 MΩ) were filled with a saturated solution of K_2_SO_4_. Cells were visualized under an inverted microscope (Axiovert; Zeiss), and impaled using micromanipulators (Narishige). VD4, VF and RPeD1 neurons were identified by size, plating position, and electrophysiological signature. Signals were amplified with a Neuro data dual channel intracellular recording amplifier (Neuron Data Instruments), digitized (Digidata 1440; Molecular Devices), and recorded with Axoscope 10.2 (Molecular Devices). For in situ recordings, *Lymnaea* CNS were dissected as above and briefly treated (5–10 s) with focally applied Protease Type XIV (Sigma-Aldrich) to soften and remove the inner sheath. Isolated CNS were maintained in normal *Lymnaea* saline (in mM: 51.3 NaCl; 1.7 KCl; 4.1 CaCl_2_; 1.5 MgCl_2_; 5 HEPES; pH 7.9), and neurons were visualized under a Wild stereoscope (Wild Leitz AG), identified by size, position and color, then impaled with K_2_SO_4_-filled glass microelectrodes (resistance 20–50 MΩ) using micromanipulators (Sutter Instruments). Signals were amplified with a Neuro data amplifier, digitized (NI DAQPad; National Instruments), and recorded using custom ElectroLite software.

To determine ACh release characteristics from VD4, the mean amplitude of 5 consecutive PSPs, recorded in VF or RPeD1 neurons at a holding potential of − 100 mV was measured in response to single action potentials elicited by brief depolarizing current injection in VD4. Peptidergic transmission via FMRF neuropeptides was isolated pharmacologically using an AChR antagonist cocktail containing 5 μM methyllcaconitine (MLA), 10 μM tubocurarine (TC), and 20 μM tetraethylammonium (TEA), incubation time 5–10 min^87^. To determine FMRF neuropeptide release characteristics from VD4, the excitation of VF at holding potentials of − 60 mV in vitro or − 70 mV in situ (below firing threshold), or inhibition of RPeD1 at − 50 mV in vitro or − 60 mV in situ (at firing threshold), was measured in response to a ~ 4 s burst elicited by depolarizing current injection in VD4. These holding potentials were used for in vitro and in situ recordings because ionic differences between the external recording solutions shift neuronal firing thresholds. Peptidergic transmission was deemed to have occurred if stimulation of VD4 resulted in the induction of action potentials in VF (exhibits an excitatory response to FMRF neuropeptides from VD4) or the cessation of firing and hyperpolarization of RPeD1 (exhibits an inhibitory response to FMRF neuropeptides from VD4). To determine DA release characteristics from RPeD1, the inhibition of VD4 at a holding potential of − 50 mV was measured in response to a ~ 4 s burst elicited by depolarizing current injection in RPeD1. Dopaminergic transmission was deemed to have occurred if stimulation of RPeD1 resulted in the cessation of firing and hyperpolarization of VD4 (exhibits an inhibitory response to DA from RPeD1). In presynaptic neurons neither the duration nor the number of action potentials per burst were significantly different amongst experimental conditions (see Supplementary Information). Data analysis was performed using AxoScope 10.3 (Molecular Devices).

### Chemicals

TC, MLA, TEA and pertussis toxin (PTX) were purchased from Sigma-Aldrich. AA, Chelerythrine chloride (Ch Cl^-^), 3-(2,4-Dichlorophenyl)-4-(1-methyl-1*H*-indol-3-yl)-1*H*-pyrrole-2,5-dione (SB 216763) and Tocrisolve 100 were purchased from Tocris. 5,8,11,14-Eicosatetraynoic acid (ETYA) was purchased from Santa Cruz Biotech. Drugs were dissolved in dimethyl sulfoxide (DMSO) or DM according to solubility, and then added to culture media shortly after neurons were plated (~ 1 h). The next day, three washes with DM followed by replacement with fresh DM or CM was performed to remove drugs prior to experiments (~ 1 h). 0.1% DMSO and Tocrisolve 100 vehicle controls were performed for all experiments, as appropriate.

### Experimental design and statistical analysis

All reported results are derived from ≥ 2 independent experiments, to ensure reliability and replicability of the data. Sample sizes were limited by uncontrollable factors inherent to the cell culturing techniques used. Data analysis was performed blinded by acquisition file number. Statistical analyses were performed using SPSS Statistics v26 (IBM). Data sets were assessed with Shapiro–Wilk test for normality, and parametric (*P* > 0.05) or non-parametric (*P* < 0.05) statistical tests were performed as appropriate. Non-parametric data sets were analyzed with Wilcoxon Signed Ranks tests, Mann–Whitney U tests, or Kruskal–Wallis tests. Parametric data sets were analyzed with Student’s Paired or Independent Samples T-tests, or One-way ANOVA with Games-Howell (unequal variance; Levene statistic *P* < 0.05) or Tukey’s HSD (equal variance; Levene statistic *P* > 0.05) post hoc tests, as appropriate (see Supplementary Information).

## Supplementary information

Supplementary Information.

## Data Availability

All data generated or analyzed during this study are included in this published article and its Supplementary Information file. Materials generated during the current study are available from the corresponding authors on reasonable request.
