# Supplementary material for: Neurotrophic factors and target-specific retrograde signaling interactions define the specificity of classical and neuropeptide cotransmitter release at identified Lymnaea synapses
Source: Sci Rep. 2020 Aug 11;10:13526. doi: 10.1038/s41598-020-70322-5 (PMC7419297; doi:10.1038/s41598-020-70322-5)
Supplement: Supplementary file 1 — Supplementary Information. [file 41598_2020_70322_MOESM1_ESM.pdf]

SUPPLEMENTARY INFORMATION FOR

Neurotrophic factors and target-specific retrograde signaling interactions define the specificity of classical and neuropeptide cotransmitter release at identified *Lymnaea* synapses

AUTHORS

Angela M. Getz, Tara A. Janes, Frank Visser, Wali Zaidi & Naweed I. Syed

This File Contains:

Supplementary Data:

Tables S1 to S9

Figures S2 to S6

Supplementary Experimental Procedures:

Table S10

Supplementary References

SUPPLEMENTARY DATA

Table S1. Related to Figure 1. Cotransmission and Isolated FMRF Synaptic Transmission at VD4-VF and VD4-RPeD1 Synapses in Intact Brain Preparation *ex vivo*

Metric [1]	Control (N=11)	AChR-I (N=11)	*P*
VD4 (Duration of Burst)	4.60 ± 0.19 (s)	5.14 ± 0.28 (s)	0.136 [2]
VD4 (# of Action Potentials Fired)	41.45 ± 2.58	41.05 ± 3.61	0.910 [2]
VF (# of Action Potentials Induced)	3.00 ± 0.70	2.82 ± 0.68	0.813 [2]
RPeD1 (Duration of Inhibition) [4]	8.57 ± 1.69 (s)	1.77 ± 0.86 (s)	0.003 [3]

1. Synaptic responses were measured in the absence (control; *Lymnaea* saline) or presence of AChR antagonists (AChR-I; *Lymnaea* saline with 5 µM MLA, 10 µM TC, 20 µM TEA), excitation in VF was measured at a holding potential of -70 mV, inhibition in RPeD1 was measured at -60 mV
2. Paired Samples T-test, 2-sided
3. Wilcoxon Signed Ranks test, 2-sided
4. RPeD1 inhibition results from ACh + FMRF cotransmission in control conditions

Table S2A. Related to Figure 2. Cotransmission and Isolated FMRF Synaptic Transmission at VD4-VF and VD4-RPeD1 Soma-Soma Synapses *in vitro*

VD4-VF Metrics [1]	Treatment	CM (N=9)	DM (N=10)	*F; P* [2,3]	*P* [4]	*P* [5]	*P* [6]
VD4 (Duration of Burst) (s)	Control	3.84 ± 0.22	4.02 ± 0.23	0.227; 0.877 [2]	0.906 0.434 [b]	0.993 0.361 [b]	0.959
	AChR-I	4.09 ± 0.38	4.12 ± 0.21				1.000
VD4 (# of Action Potentials Fired)	Control	17.94 ± 1.41	17.05 ± 0.98	0.613; 0.611 [2]	0.694 0.153 [b]	0.899 0.689 [b]	0.958
	AChR-I	15.94 ± 1.33	15.85 ± 1.30				1.000
VF (# of Action Potentials Induced) (s)	Control	3.56 ± 2.46	0.30 ± 0.20	24.215; <0.001 [3]	0.505 0.514 [c]	0.950 0.785 [c]	0.002
	AChR-I	5.06 ± 1.22	0.35 ± 0.24				<0.001
**VD4-RPeD1 Metrics [1]**	**Treatment**	**CM (N=10)**	**DM (N=11)**	***F; P* [2,3]**	***P* [4]**	***P* [5]**	***P* [6]**
VD4 (Duration of Burst) (s)	Control	3.89 ± 0.28	3.87 ± 0.25	0.417; 0.937 [3]	0.705 0.568 [b]	0.554 0.417 [b]	0.833
	AChR-I	4.13 ± 0.36	4.14 ± 0.37				0.973
VD4 (# of Action Potentials Fired)	Control	19.20 ± 1.57	20.14 ± 1.84	1.129; 0.350 [2]	0.808 0.288 [b]	0.390 0.078 [b]	0.975
	AChR-I	17.15 ± 1.67	16.64 ± 1.19				0.996
RPeD1 (Duration of Inhibition) (s) [7]	Control	8.54 ± 1.39	5.52 ± 0.70	13.416; 0.004 [3]	<0.001 0.005 [c]	0.683 0.213 [c]	0.151
	AChR-I	2.19 ± 0.77	7.51 ± 1.83				0.010

1. Cotransmission-mediated synaptic responses were measured in control conditions (CM or DM), FMRF-mediated synaptic responses were measured in the presence of AChR antagonists (AChR-I; CM or DM with 5 µM MLA, 10 µM TC, 20 µM TEA), excitation in VF was measured at a holding potential of -60 mV, inhibition in RPeD1 was measured at -50 mV
2. One-way ANOVA, Tukey's HSD *post hoc* test
3. Independent Samples Kruskal-Wallis test
4. Statistical significance, CM control relative to CM AChR-I
   4b: Paired Samples T-test, 2-sided; 4c: Wilcoxon Signed Ranks test, 2-sided
5. Statistical significance, DM control relative to DM AChR-I
   5b: Paired Samples T-test, 2-sided; 5c: Wilcoxon Signed Ranks test, 2-sided
6. Statistical significance, CM control relative to DM control (top), and CM AChR-I relative to DM AChR-I (bottom)
7. RPeD1 inhibition reflects ACh + FMRF cotransmission in control conditions

Table S2B. Related to Figure 2. ACh Synaptic Transmission and Cotransmission at VD4-VF and VD4-RPeD1 Soma-Soma Synapses *in vitro*

VD4-VF Metrics	CM (N=9)	DM (N=10)	*F; P* [2]	*P* [3]	*P* [4]
PSP Amplitude (mV) [1]	9.04 ± 1.54	9.69 ± 1.52	2.422; 0.490	0.683	0.288
Excitatory Biphasic Component (# of action potentials induced) [6]	1.44 ± 0.19	0.10 ± 0.10	26.831; <0.001	0.001	0.667
Inhibitory Biphasic Component (duration of inhibition) (s) [6]	4.66 ± 1.07 (s)	5.01 ± 0.42	8.596; 0.035	0.434	0.005
**VD4-RPeD1 Metrics**	**CM (N=10)**	**DM (N=11)**	***F; P* [2]**	***P* [3]**	***P* [5]**
PSP Amplitude (mV) [1]	11.13 ± 1.22	8.49 ± 1.54	2.422; 0.490	0.121	0.526
Excitatory Biphasic Component (# of action potentials induced) [6]	2.80 ± 0.71	0.14 ± 0.25	26.831; <0.001	<0.001	0.865
Inhibitory Biphasic Component (duration of inhibition) (s) [6,7]	8.54 ± 1.39	5.52 ± 0.70	8.596; 0.035	0.078	0.711

1. Amplitudes of ACh-mediated PSPs were measured under control conditions (CM or DM) in response to a single action potential elicited in VD4, postsynaptic VF or RPeD1 neurons were maintained at a holding potential of -100 mV; The mean amplitudes of 5 consecutive PSPs were measured per synaptic pair
2. Independent Samples Kruskal-Wallis test
3. Statistical significance, CM control relative to DM control (VD4-VF or VD4-RPeD1)
4. Statistical significance, VD4-VF control (CM) relative to VD4-RPeD1 control (CM)
5. Statistical significance, VD4-VF control (DM) relative to VD4-RPeD1 control (DM)
6. ACh-mediated biphasic (excitation followed by inhibition) or inhibitory responses were measured in CM or DM, VF was held at -60 mV and RPeD1 was held at -50 mV
7. Biphasic inhibition reflects ACh + FMRF cotransmission in VD4-RPeD1 pairs without AChR-I

Table S3A. Related to Figure 3. Cotransmission and Isolated FMRF Synaptic Transmission at VD4-VF and VD4-RPeD1 Triple-Soma Synapses *in vitro*

Triple Soma Metrics [1]	Treatment	CM (N=9)	DM (N=10)	*F; P* [2,3]	*P* [4]	*P* [5]	*P* [6]
VD4 (Duration of Burst) (s)	Control	4.07 ± 0.21	4.46 ± 0.26	7.573; 0.056 [2]	0.401	0.086	0.270
	AChR-I	4.71 ± 0.38	5.17 ± 0.25		0.139 [c]	0.173 [c]	0.121
VD4 (# of Action Potentials Fired)	Control	18.22 ± 2.17	18.89 ± 1.96	0.389; 0.762 [3]	0.932	0.997	0.997
	AChR-I	16.33 ± 2.77	19.50 ± 1.86		0.614 [b]	0.930 [b]	0.734
VF (# of Action Potentials Induced) (s)	Control	3.72 ± 1.16	0.00 ± 0.00	21.092; <0.001 [2]	0.577	0.583	0.002
	AChR-I	4.44 ± 0.96	0.40 ± 0.27		0.635 [c]	0.157 [c]	0.001
RPeD1 (Duration of Inhibition) (s) [7]	Control	5.56 ± 0.89	6.64 ± 1.73	12.524; 0.006 [2]	0.009	0.624	0.956
	AChR-I	1.16 ± 0.70	6.71 ± 1.22		0.008 [c]	0.953 [c]	0.001

1. Cotransmission-mediated synaptic responses were measured in control conditions (CM or DM), FMRF-mediated synaptic responses were measured in the presence of AChR antagonists (AChR-I; CM or DM with 5 μM MLA, 10 μM TC, 20 μM TEA), excitation in VF was measured at a holding potential of -60 mV, inhibition in RPeD1 was measured at -50 mV
2. Independent Samples Kruskal-Wallis test
3. One-way ANOVA, Tukey's HSD *post hoc* test
4. Statistical significance, CM control relative to CM AChR-I
   4b: Paired Samples T-test, 2-sided; 4c: Wilcoxon Signed Ranks test, 2-sided
5. Statistical significance, DM control relative to DM AChR-I
   5b: Paired Samples T-test, 2-sided; 5c: Wilcoxon Signed Ranks test, 2-sided
6. Statistical significance, CM control relative to DM control (top), and CM AChR-I relative to DM AChR-I (bottom)
7. RPeD1 inhibition reflects ACh + FMRF cotransmission in control conditions

Table S3B. Related to Figure 3. ACh Synaptic Transmission and Cotransmission at VD4-VF and VD4-RPeD1 Triple-Soma Synapses *in vitro*

VD4-VF Metrics	CM (N=9)	DM (N=10)	*F; P* [2,3]	*P* [4]	*P* [5]
PSP Amplitude (mV) [1]	3.71 ± 0.61	3.09 ± 0.46	15.386; 0.002 [2]	0.623	0.012
Excitatory Biphasic Component (# of action potentials induced) [7]	0.72 ± 0.32	0.06 ± 0.06	9.007; 0.029 [2]	0.054	0.659
Inhibitory Biphasic Component (duration of inhibition) (s) [7]	7.21 ± 0.87	4.41 ± 0.90	1.141; 0.347 [3]	0.154	0.561
VD4-RPeD1 Metrics	CM (N=9)	DM (N=10)	*F; P* [2,3]	*P* [4]	*P* [6]
PSP Amplitude (mV) [1]	8.55 ± 1.71	7.36 ± 0.80	15.386; 0.002 [2]	1.000	0.003
Excitatory Biphasic Component (# of action potentials induced) [7]	1.22 ± 0.43	0.11 ± 0.11	9.007; 0.029 [2]	0.023	0.925
Inhibitory Biphasic Component (duration of inhibition) (s) [7,8]	5.56 ± 0.89	6.64 ± 1.73	1.141; 0.347 [3]	0.944	0.672

1. Amplitudes of ACh-mediated PSPs were measured under control conditions (CM or DM) in response to a single action potential elicited in VD4, postsynaptic VF or RPeD1 neurons were maintained at a holding potential of -100 mV; The mean amplitudes of 5 consecutive PSPs were measured per synaptic pair
2. Independent Samples Kruskal-Wallis test
3. One-way ANOVA, Games-Howell *post hoc* test
4. Statistical significance, CM control relative to DM control (VD4-VF or VD4-RPeD1)
5. Statistical significance, VD4-VF control (CM) relative to VD4-RPeD1 control (CM)
6. Statistical significance, VD4-VF control (DM) relative to VD4-RPeD1 control (DM)
7. ACh-mediated biphasic (excitation followed by inhibition) or inhibitory responses were measured in CM or DM, VF was held at -60 mV and RPeD1 was held at -50 mV
8. Biphasic inhibition reflects ACh + FMRF cotransmission in VD4-RPeD1 pairs without AChR-I

Table S4. Related to Figure 4. DA Synaptic Transmission at RPeD1-VD4 Soma-Soma and Triple-Soma Synapses

RPeD1-VD4	RPeD1 (Duration of Burst)	*F; P* [2]	*P* [3]	N
CM Soma-Soma	5.69 ± 0.36	1.638; 0.201	-	7
DM Soma-Soma	4.90 ± 0.34		0.411	10
CM Triple-Soma	5.38 ± 0.35		0.943	7
DM Triple-Soma	5.44 ± 0.18		0.987	10
RPeD1-VD4	RPeD1 (# of Action Potentials Fired)	*F; P* [2]	*P* [4]	N
CM Soma-Soma	18.50 ± 2.06	1.413; 0.258	-	7
DM Soma-Soma	20.95 ± 2.28		0.855	10
CM Triple-Soma	25.14 ± 1.24		0.081	7
DM Triple-Soma	21.05 ± 2.16		0.828	10
RPeD1-VD4[1]	VD4 (Duration of Inhibition) (s)	*F; P* [2]	*P* [3]	N
CM Soma-Soma	9.50 ± 0.82	5.522; 0.004	-	7
DM Soma-Soma	4.26 ± 1.19		0.010	10
CM Triple-Soma	4.50 ± 1.25		0.028	7
DM Triple-Soma	3.70 ± 0.93		0.004	10

1. DA-mediated synaptic responses were measured by inhibition in VD4, at a holding potential of -50 mV
2. One-way ANOVA, statistical significance relative to CM Soma-Soma
3. Tukey's HSD *post hoc* test
4. Games-Howell *post hoc* test

Table S5A. Related to Figure 5. Somatic VD4 FMRF Neuropeptide Fluorescence

Culture Condition	Mean Fluorescence (AU)	*P* [1]	N
Unpaired Neurons			
CM VD4	151.40 ± 9.09	(F=0.899; *P*=0.512)	8
DM VD4	142.76 ± 7.49	0.998	11
Axon-Axon Paired Neurons			
CM VD4 (VF)	142.19 ± 10.24	0.998	10
CM VD4 (RPeD1)	146.25 ± 12.30	1.000	8
DM VD4 (VF)	164.35 ± 8.04	0.981	10
DM VD4 (RPeD1)	159.37 ± 5.48	0.999	9
Triple-Axon Paired Neurons			
CM VD4 (VF, RPeD1)	144.83 ± 8.65	1.000	10
DM VD4 (VF, RPeD1)	158.87 ± 10.21	0.999	13

1. One-way ANOVA, statistical significance relative to unpaired CM VD4, Tukey's HSD *post hoc* test

Table S5B. Related to Figure 5. Synaptic VD4 FMRF Neuropeptide Fluorescence

Culture Condition	Mean Fluorescence (AU)	*P* [1]	N
Axon-Axon Paired Neurons			
CM VD4 (VF)	70.39 ± 5.10	(F=9.795; *P*<0.001)	10
CM VD4 (RPeD1)	112.85 ± 11.45	0.031	8
DM VD4 (VF)	122.41 ± 13.18	0.015	10
DM VD4 (RPeD1)	65.81 ± 3.97	0.892	9
Triple-Axon Paired Neurons			
CM VD4 (VF)	79.63 ± 7.03	(F=9.979; *P*<0.001)	10
CM VD4 (RPeD1)	119.87 ± 9.22	0.008	
DM VD4 (VF)	120.62 ± 8.18	0.004	13
DM VD4 (RPeD1)	76.05 ± 6.68	0.988	

1. One-way ANOVA, statistical significance relative to CM VD4 (VF), Games-Howell *post hoc* test (axon-axon) or Tukey's HSD *post hoc* test (triple-axon)

Table S6A. Related to Figure 6. FMRF Synaptic Transmission at VD4-VF and VD4-RPeD1 Soma-Soma Synapses

VD4-VF Metrics [1]	CM+H_2O vehicle (N=9)	CM+*L*-SYP mRNA (N=7)	*P*
VD4 (Duration of Burst)	4.21 ± 0.47 (s)	4.76 ± 0.37 (s)	0.390 [2]
VD4 (# of Action Potentials Fired)	17.61 ± 2.00	17.79 ± 1.90	0.952 [2]
VF (# of Action Potentials Induced)	5.22 ± 0.83	0.71 ± 0.24	<0.001 [3]
VD4-RPeD1 Metrics [1]	DM+H_2O vehicle (N=11)	DM+*L*-SYP mRNA (N=9)	*P*
VD4 (Duration of Burst)	4.23 ± 0.27 (s)	4.25 ± 0.20 (s)	0.652 [2]
VD4 (# of Action Potentials Fired)	17.73 ± 1.21	16.89 ± 1.38	0.936 [2]
RPeD1 (Duration of Inhibition)	6.95 ± 1.81 (s)	1.87 ± 0.85 (s)	0.025 [3]

1. FMRF-mediated synaptic responses were measured in the presence of AChR antagonists (CM or DM with 5 µM MLA, 10 µM TC, 20 µM TEA), excitation in VF was measured at a holding potential of -60 mV, inhibition in RPeD1 was measured at a holding potential of -50 mV.
2. Independent Samples T-test, 2-sided
3. Mann-Whitney U test, 2-sided

Table S6B. Related to Figure 6. ACh Synaptic Transmission at VD4-VF and VD4-RPeD1 Soma-Soma Synapses

Culture Condition	Mean PSP Amplitude (mV)	*P* [1]	N
CM VD4-VF			
VD4 + H_2O vehicle	9.53 ± 1.39	0.613	10
VD4 + *L*-SYP mRNA	8.27 ± 1.82		14
DM VD4-RPeD1			
VD4 + H_2O vehicle	6.99 ± 1.69	0.898	9
VD4 + *L*-SYP mRNA	6.70 ± 1.43		9

1. Independent Samples T-test, 2-sided

Table S6C. Related to Figure 6. Somatic and Synaptic VD4 FMRF Neuropeptide Fluorescence

Culture Condition	Mean Somatic Fluorescence (AU)	*P* [1]	N
CM VD4 + H_2O vehicle	376.42 ± 78.20	0.017	6
CM VD4 + *L*-SYP mRNA	643.64 ± 45.52		7
Culture Condition	Mean Synaptic Fluorescence (AU)	*P* [1]	N
CM VD4 (H_2O vehicle) - VF	205.75 ± 13.97	0.028	6
CM VD4 (*L*-SYP mRNA) - VF	388.74 ± 65.39		7

1. Independent Samples T-test, 2-sided

Table S6D. Related to Figure 6. Somatic and Synaptic SYP-mCherry Fluorescence

Culture Condition	Mean Somatic Fluorescence (AU)	*P* [1]	N
CM VD4 + *L*-SYP-mCherry mRNA	131.93 ± 31.70	(F=9.255; *P*=0.015)	3
CM VF	34.31 ± 4.75	0.023	
CM RPeD1	33.55 ± 3.23	0.022	
Culture Condition	Mean Synaptic Fluorescence (AU)	*P* [2]	N
CM VD4 (*L*-SYP-mCherry) - VF	74.31 ± 11.81	<0.001	3
CM VD4 (*L*-SYP-mCherry) - RPeD1	189.34 ± 11.93		

2. One-way ANOVA, Tukey's HSD *post hoc* test, statistical significance relative to CM VD4 + *L*-SYP-mCherry mRNA
3. Independent Samples T-test, 2-sided

Table S7A. Related to Figure 7. FMRF Synaptic Transmission at VD4-VF Soma-Soma Synapses

VD4-VF [1]	VD4 (Duration of Burst)	*F; P* [2]	*P* [3]	N
CM + vehicle	4.39 ± 0.18	1.936; 0.139	-	10
CM + SB216763	4.77 ± 0.21		0.493	11
DM + vehicle	4.41 ± 0.15		1.000	11
DM + SB216763	4.17 ± 0.17		0.815	12
VD4-VF [1]	VD4 (# of Action Potentials Fired)	*F; P* [2]	*P* [3]	N
CM + vehicle	20.62 ± 1.95	1.610; 0.202	-	10
CM + SB216763	16.45 ± 1.68		0.201	11
DM + vehicle	18.27 ± 0.84		0.671	11
DM + SB216763	19.79 ± 1.13		0.977	12
VD4-VF [1]	VF (# of Action Potentials Induced)	*F; P* [4]	*P* [5]	N
CM + vehicle	3.49 ± 0.78	11.769; 0.008	0.114	10
CM + SB216763	1.77 ± 0.70			11
DM + vehicle	0.27 ± 0.19		0.013	11
DM + SB216763	2.29 ± 0.55			12

1. FMRF-mediated synaptic responses were measured in the presence of AChR antagonists (CM or DM with 5 µM MLA, 10 µM TC, 20 µM TEA), excitation in VF was measured at a holding potential of -60 mV
2. One-way ANOVA
3. Tukey's HSD *post hoc* test, relative to CM + vehicle
4. Independent Samples Kruskal-Wallis test
5. Pairwise comparisons, relative to CM or DM + vehicle

Table S7B. Related to Figure 7. FMRF Synaptic Transmission at VD4-RPeD1 Soma-Soma Synapses

VD4-RPeD1 [1]	VD4 (Duration of Burst)	*F; P* [2]	*P* [3]	N
CM + vehicle	4.36 ± 0.17	0.373; 0.773	-	9
CM + SB216763	4.62 ± 0.23		0.747	11
DM + vehicle	4.53 ± 0.21		0.927	8
DM + SB216763	4.45 ± 0.10		0.984	12
VD4-RPeD1 [1]	VD4 (# of Action Potentials Fired)	*F; P* [2]	*P* [3]	N
CM + vehicle	16.06 ± 1.76	0.740; 0.535	-	9
CM + SB216763	18.68 ± 1.69		0.674	11
DM + vehicle	19.56 ± 1.23		0.511	8
DM + SB216763	17.75 ± 1.61		0.879	12
VD4-RPeD1 [1]	RPeD1 (Duration of Inhibition)	*F; P* [4]	*P* [5]	N
CM + vehicle	0.37 ± 0.37	16.209; 0.001	0.003	9
CM + SB216763	6.65 ± 1.90			11
DM + vehicle	5.02 ± 1.73		0.009	8
DM + SB216763	0.44 ± 0.29			12

1. FMRF-mediated synaptic responses were measured in the presence of AChR antagonists (CM or DM with 5 µM MLA, 10 µM TC, 20 µM TEA), inhibition in RPeD1 was measured at a holding potential of -50 mV
2. One-way ANOVA
3. Tukey's HSD *post hoc* test, relative to CM + vehicle
4. Independent Samples Kruskal-Wallis test
5. Pairwise comparisons, relative to CM or DM + vehicle

Table S8A. Related to Figure 8. FMRF Synaptic Transmission at VD4-VF Soma-Soma Synapses

VD4-VF [1]	VD4 (Duration of Burst)	*F; P* [2]	*P* [3]	N
DM + vehicle	4.16 ± 0.12	1.739; 0.188	-	19
DM + AA	4.43 ± 0.20		0.388	13
DM + ETYA	4.52 ± 0.14		0.204	13
VD4-VF [1]	VD4 (# of Action Potentials Fired)	*F; P* [2]	*P* [3]	N
DM + vehicle	18.08 ± 0.67	1.515; 0.232	-	19
DM + AA	17.04 ± 0.55		0.565	13
DM + ETYA	18.96 ± 0.93		0.662	13
VD4-VF [1]	VF (# of Action Potentials Induced)	*F; P* [4]	*P* [5]	N
DM + vehicle	0.16 ± 0.09	15.775; <0.001	-	19
DM + AA	2.92 ± 0.81		<0.001	13
DM + ETYA	0.07 ± 0.05		0.889	13

1. FMRF-mediated synaptic responses were measured in the presence of AChR antagonists (DM with 5 µM MLA, 10 µM TC, 20 µM TEA), excitation in VF was measured at a holding potential of -60 mV,
2. One-way ANOVA
3. Tukey's HSD *post hoc* test, relative to DM + vehicle
4. Independent Samples Kruskal-Wallis test
5. Pairwise comparisons, relative to DM + vehicle

Table S8B. Related to Figure 8. FMRF Synaptic Transmission at VD4-RPeD1 Soma-Soma Synapses

VD4-RPeD1 [1]	VD4 (Duration of Burst)	*F; P* [2]	*P* [3]	N
DM + vehicle	4.46 ± 0.14	1.298; 0.283	-	19
DM + AA	4.67 ± 0.12		0.592	16
DM + ETYA	4.81 ± 0.20		0.263	15
VD4-RPeD1 [1]	VD4 (# of Action Potentials Fired)	*F; P* [2]	*P* [3]	N
DM + vehicle	17.50 ± 1.25	0.698; 0.502	-	19
DM + AA	17.00 ± 1.08		0.948	16
DM + ETYA	18.93 ± 1.05		0.656	15
VD4-RPeD1 [1]	RPeD1 (Duration of Inhibition)	*F; P* [4]	*P* [5]	N
DM + vehicle	4.95 ± 1.31	8.668; 0.013	-	19
DM + AA	3.98 ± 1.00		0.933	16
DM + ETYA	0.82 ± 0.49		0.010	15

1. FMRF-mediated synaptic responses were measured in the presence of AChR antagonists (DM with 5 µM MLA, 10 µM TC, 20 µM TEA), inhibition in RPeD1 was measured at a holding potential of -50 mV
2. One-way ANOVA
3. Tukey's HSD *post hoc* test, relative to DM + vehicle
4. Independent Samples Kruskal-Wallis test
5. Pairwise comparisons, relative to DM + vehicle

Table S9A. Related to Figure 9. FMRF Synaptic Transmission at VD4-RPeD1 Soma-Soma Synapses

VD4-RPeD1 [1]	VD4 (Duration of Burst)	*P* [2]	N
DM + vehicle	4.26 ± 0.10	0.441	11
DM + Chelerythrine Cl$^-$	4.42 ± 0.19		8
VD4-RPeD1 [1]	VD4 (# of Action Potentials Fired)	*P* [2]	N
DM + vehicle	17.77 ± 1.41	0.223	11
DM + Chelerythrine Cl$^-$	15.31 ± 1.20		8
VD4-RPeD1 [1]	RPeD1 (Duration of Inhibition)	*P* [3]	N
DM + vehicle	7.23 ± 1.91	0.007	11
DM + Chelerythrine Cl$^-$	0.60 ± 0.60		8

1. FMRF-mediated synaptic responses were measured in the presence of AChR antagonists (DM with 5 µM MLA, 10 µM TC, 20 µM TEA), inhibition in RPeD1 was measured at a holding potential of -50 mV
2. Independent Samples T-test, 2-sided
3. Mann-Whitney U test, 2-sided

Table S9B. Related to Figure 9. FMRF Synaptic Transmission at VD4-VF Soma-Soma Synapses

VD4-VF [1]	VD4 (Duration of Burst)	*F; P* [2]	*P* [3]	N
DM + vehicle	4.26 ± 0.09 *	1.084; 0.363	-	28
DM + Chelerythrine Cl$^-$	4.33 ± 0.05		0.978	10
DM + AA	4.43 ± 0.20 **		0.722	13
DM + AA + Chelerythrine Cl$^-$	4.54 ± 0.13		0.328	13
VD4-VF [1]	VD4 (# of Action Potentials Fired)	*F; P* [2]	*P* [3]	N
DM + vehicle	17.55 ± 0.53 *	1.186; 0.323	-	28
DM + Chelerythrine Cl$^-$	17.90 ± 0.74		0.980	10
DM + AA	17.04 ± 0.55 **		0.921	13
DM + AA + Chelerythrine Cl$^-$	16.23 ± 0.53		0.371	13
VD4-VF [1]	VF (# of Action Potentials Induced)	*F; P* [4]	*P* [5]	N
DM + vehicle	0.27 ± 0.09 *	18.152; <0.001	-	28
DM + Chelerythrine Cl$^-$	0.15 ± 0.11		0.706	10
DM + AA	2.92 ± 0.81 **		<0.001	13
DM + AA + Chelerythrine Cl$^-$	0.08 ± 0.08		0.312	13

1. FMRF-mediated synaptic responses were measured in the presence of AChR antagonists (DM with 5 µM MLA, 10 µM TC, 20 µM TEA), excitation in VF was measured at a holding potential of -60 mV
2. One-way ANOVA
3. Tukey's HSD *post hoc* test, relative to DM + vehicle
4. Independent-Samples Kruskal-Wallis test
5. Pairwise comparisons, relative to DM + vehicle

* Data in part repeated from Table S8A

** Data repeated from Table S8A

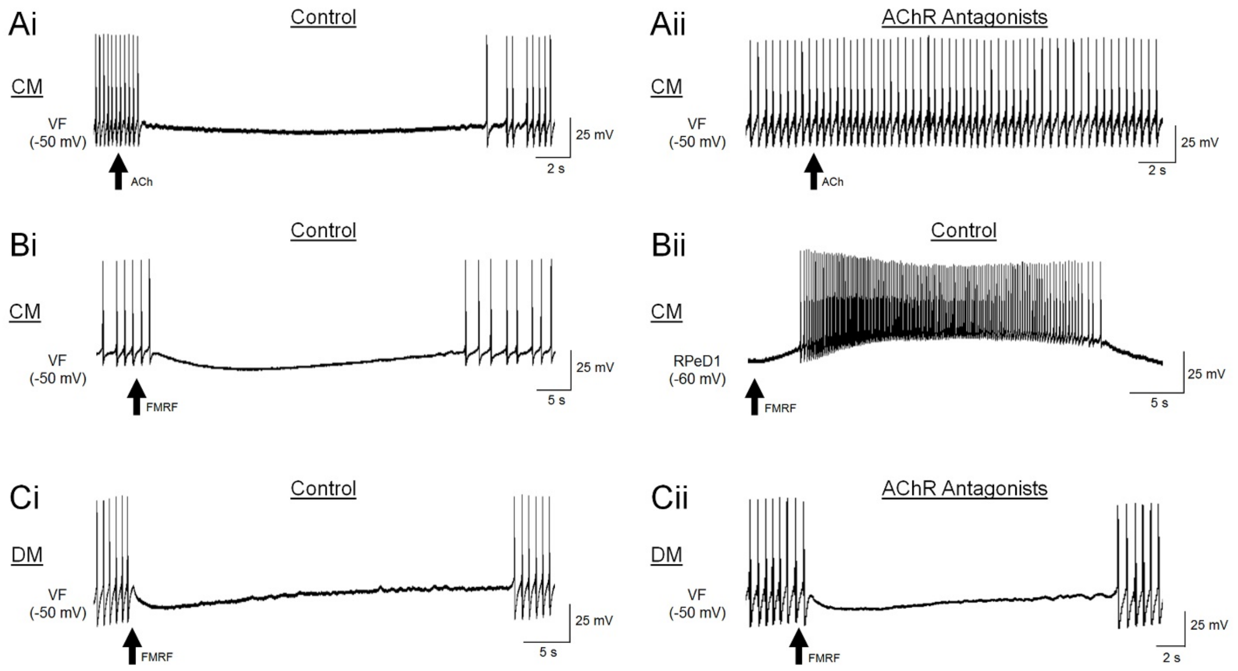

Supplementary Figure S2A: Postsynaptic receptor expression profiles

(**A**). VD4-VF were cultured in a soma-soma configuration in CM. Pressure application of 1 µM ACh (arrows; 250 ms pulse, 10 PSI, tip opening ~ 1-5 µm) elicits an inhibitory response in VF (**i**), which is eliminated in the presence of the AChR antagonist cocktail (**ii**; 5 µM MLA, 10 µM TC; 20 µM TEA). (**B**). FMRFamide heptapeptides and tetrapeptides elicit opposite responses in the same cell, and FMRFamide heptapeptides or tetrapeptides can also elicit opposite responses in different cells, through cell-specific coupling of the receptors to different G protein complexes[1,2]. As FMRFamide heptapeptides are not commercially available, we used the FMRFamide tetrapeptide (Phe-Met-Arg-Phe-NH_2) to evaluate the postsynaptic responses of VF and RPeD1 to FMRFa neuropeptides, and whether this response is influenced by the presence or absence of NTFs. VF and RPeD1 were paired in a soma-soma configuration with VD4 and cultured in CM. Pressure application of 1 mM FMRFa tetrapeptide (arrows; 250 ms pulse, 10 PSI, tip opening ~ 1-5 µm) elicits inhibition in VF neurons (**i**) and excitation in RPeD1 neurons (**ii**). Note that these responses to the FMRFamide tetrapeptide are opposite of the postsynaptic responses to the FMRFa heptapeptides released by VD4 (G/SDPFLRFamide; VF, excitatory; RPeD1, inhibitory; see Fig. 2)[3]. (**C**). NTFs do not change the profiles of postsynaptic responses to FMRFa neuropeptides. Pressure application of 1 mM FMRFamide tetrapeptide (arrows; 250 ms pulse, 10 PSI, tip opening ~ 1-5 µm) also elicits inhibition in VF cultured with VD4 in DM (**i**). The response to FMRFa is unaffected by the AChR antagonist cocktail (**ii**). N≥3 each.

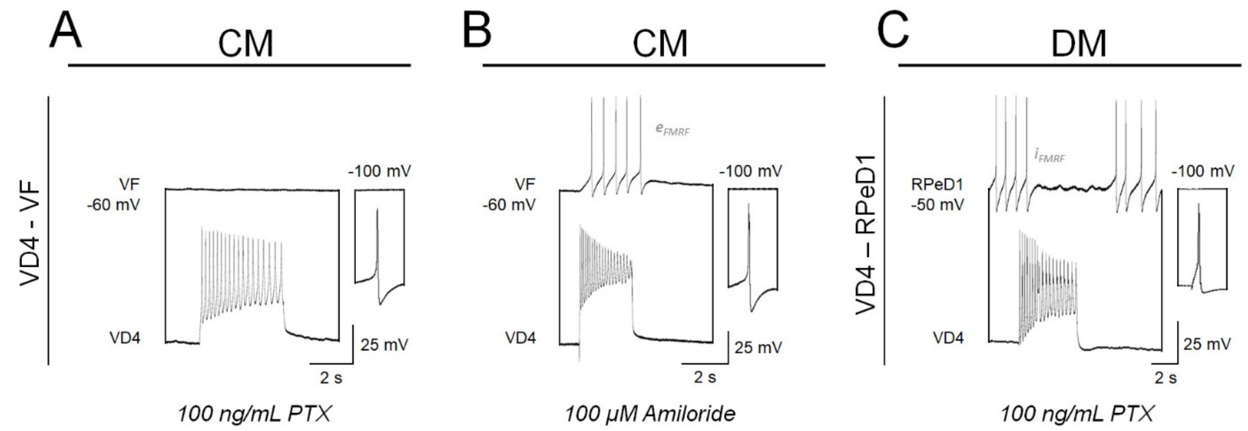

Supplementary Figure S2B: FMRF neuropeptide GPCRs are differentially coupled in VF and RPeD1

(**A**). The excitatory peptidergic postsynaptic response of VF is inhibited by 100 ng/mL pertussis toxin (PTX, $G\alpha_{i/o}$ inhibitor) at VD4-VF synapses cultured in CM and recorded in the presence of AChR antagonists (5 μM MLA, 10 μM TC, 20 μM TEA). N=3. (**B**). The excitatory peptidergic postsynaptic response of VF is unaffected by 100 μM amiloride (FMRFamide-gated sodium channel inhibitor) at VD4-VF synapses cultured in CM and recorded in the presence of AChR antagonists, confirming the role of FMRF neuropeptide-gated $G\alpha_{i/o}$ coupled GPCRs. N=3. (**C**). The inhibitory peptidergic postsynaptic response of RPeD1 is unaffected by 100 ng/mL PTX at VD4-RPeD1 synapses cultured in DM and recorded in the presence of AChR antagonists, indicating that FMRF neuropeptide receptors are differentially coupled to G protein signaling complexes in these two postsynaptic targets, and that FMRF neuropeptide-mediated postsynaptic inhibition in RPeD1 involves a PTX-insensitive G protein (non-$G\alpha_{i/o}$). N=3. Inserts show absence of ACh-PSPs to validate the isolation of peptidergic transmission by AChR antagonists (right; -100 mV holding potential).

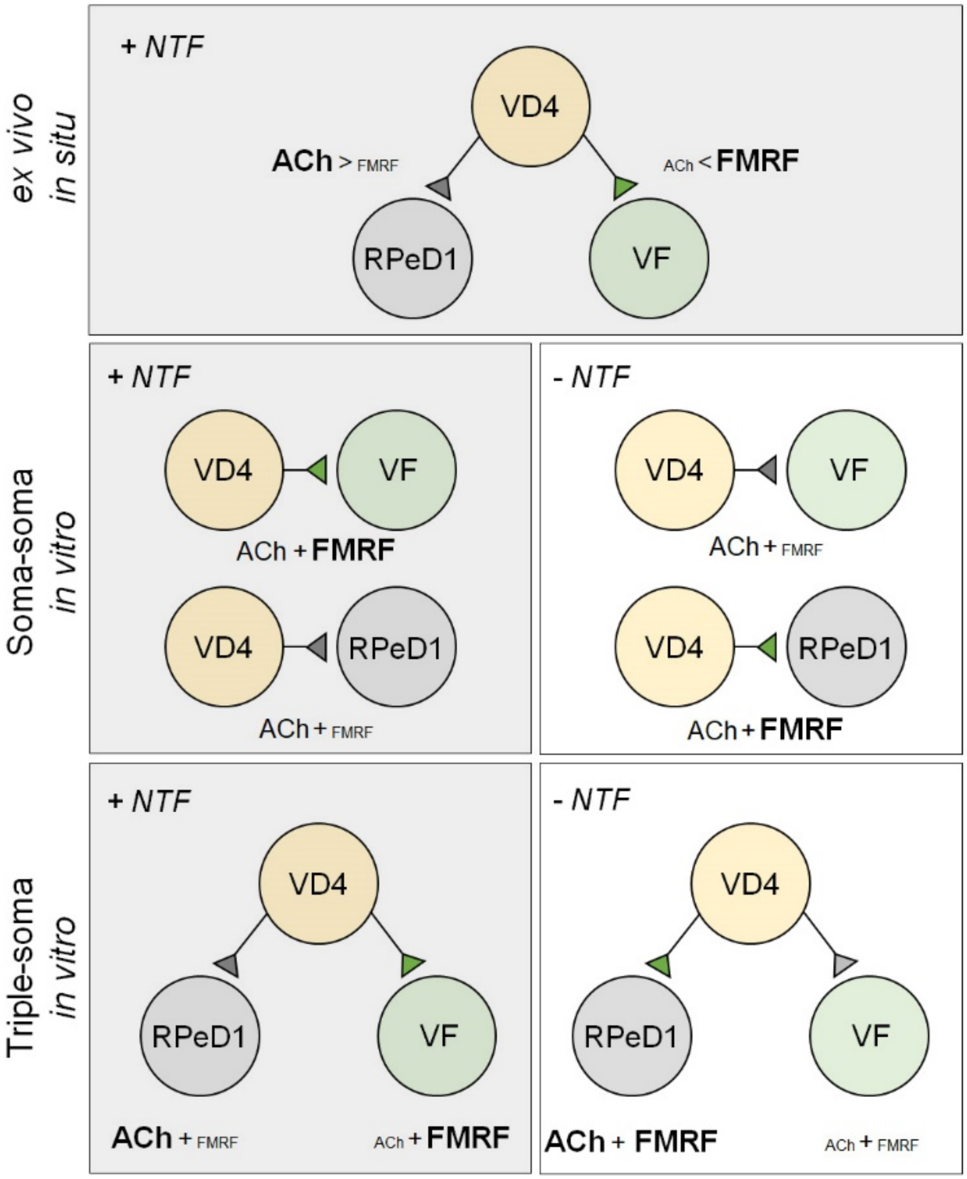

Supplementary Figure S3: Summary of context- and target-dependent regulation of cotransmission characteristics observed in experimental preparations

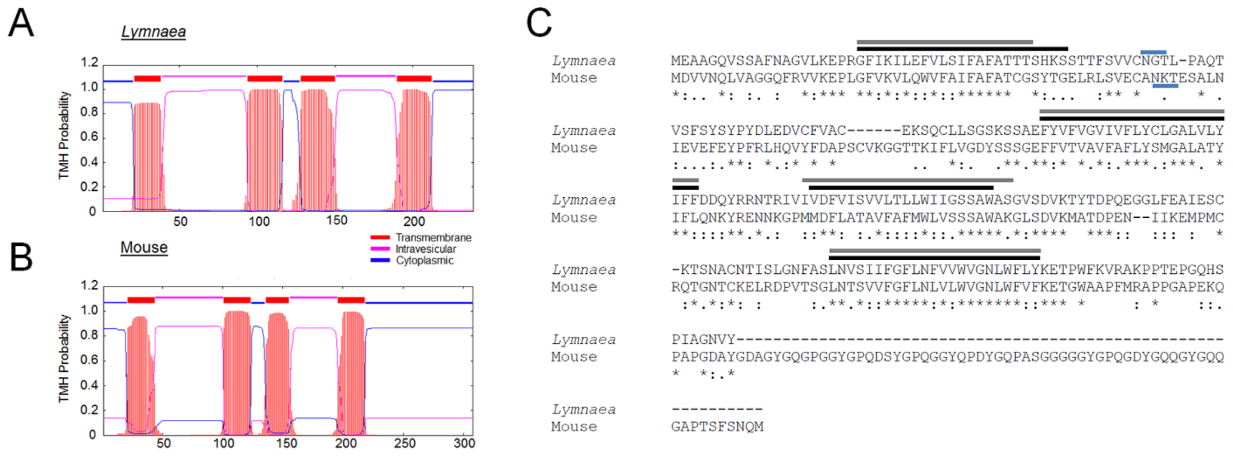

Supplementary Figure S6A: Prediction of *Lymnaea* synaptophysin transmembrane domains

(**A-B**). The *Lymnaea* Syp homologue exhibits the four transmembrane domains characteristic of the Syp family of vesicle integral membrane proteins. Amino acid residue hydrophobicity plots predict the membrane topology and location of transmembrane helices in the *Lymnaea* Syp homologue (**A**; accession no. ES572211) and mouse Syp (**B**; accession no. NM_009305). Blue indicates predicted cytoplasmic residues, red indicates predicted transmembrane residues, pink indicates predicted intravesicular residues. (TMHMM 2.0; CBS). (**C**). Protein sequence alignment of *Lymnaea* (top) and mouse (bottom) Syp (Clustal Omega; EMBL-EBI). Grey bars indicate predicted *Lymnaea* transmembrane domains, black bars indicate predicted mouse transmembrane domains. *Lymnaea* and mouse Syp contain N-glycosylation consensus sequences (N-X-T) in the first intravesicular loop (blue bars). The sequence identity of *Lymnaea* and mouse synaptophysin homologues is ~40%. *Lymnaea* Syp lacks the repetitive C-terminal domain found in mammalian Syp, however, this has also been reported for an *Aplysia* Syp homologue (Accession number XM013082245). As reported in other species, the *Lymnaea* Syp transmembrane regions are highly conserved, and intravesicular loops show higher rates of amino acid substitutions[4].

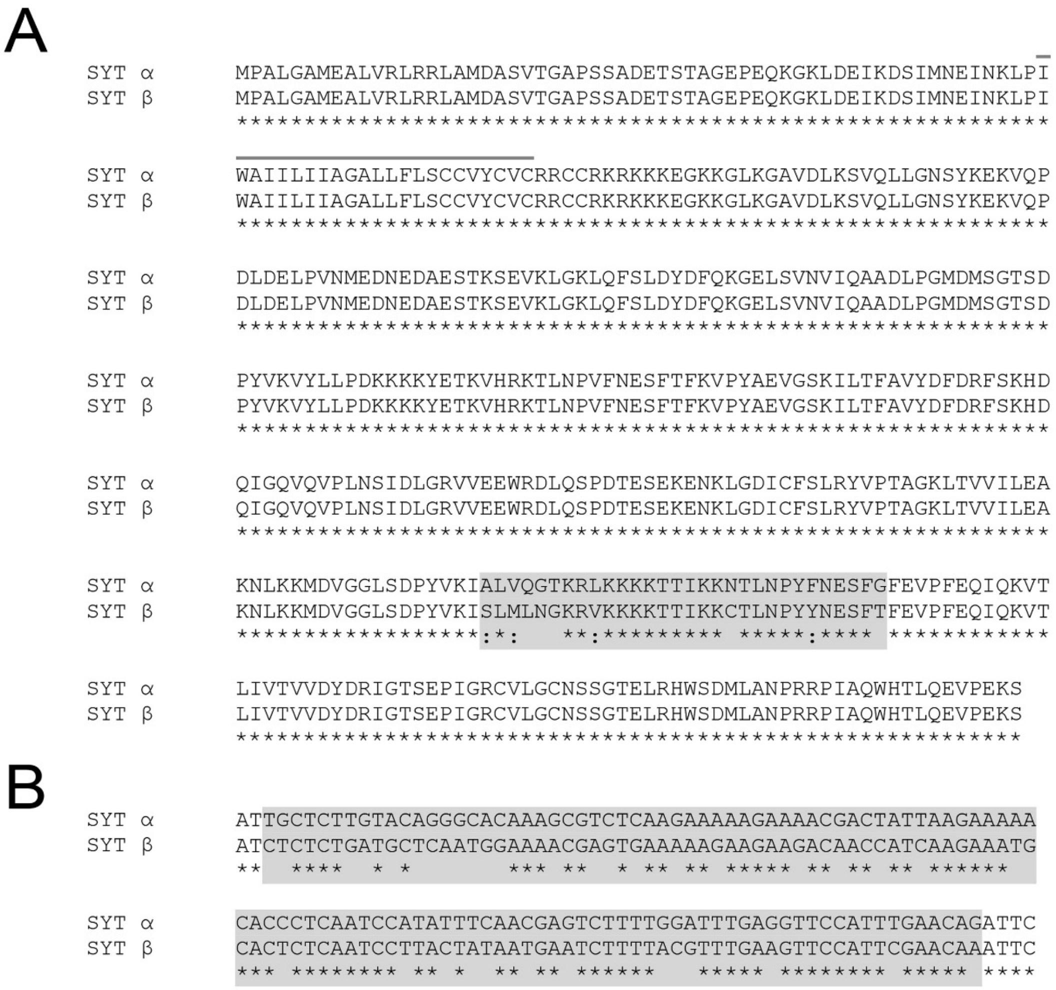

Supplementary Figure S6B: Sequences of *Lymnaea* synaptotagmin I C2B-α and C2B-β splice variants

(**A**). Protein sequence alignment of *Lymnaea* synaptotagmin I (Syt) splice variants, C2B-α (Syt-α) and C2B-β (Syt-β; accession no. AF484090). Grey bar indicates predicted transmembrane domains (see also Supplementary Figure S6C). Region of amino acid sequence variability is shaded grey. (**B**). Alternative splicing of *Lymnaea* Syt I. Region of sequence variability between *Lymnaea* Syt I splice variants is shaded grey. The alternate exon encodes 37 nucleotide changes over a region of 112 bases, which encode 9 amino acid substitutions. These substitutions in *Lymnaea* Syt are identical to the previously described *Aplysia* Syt I C2B-α and Syt I C2B-β protein isoforms that confer different affinities for v-SNARE binding and thus differential regulation of transmitter release[5,6].

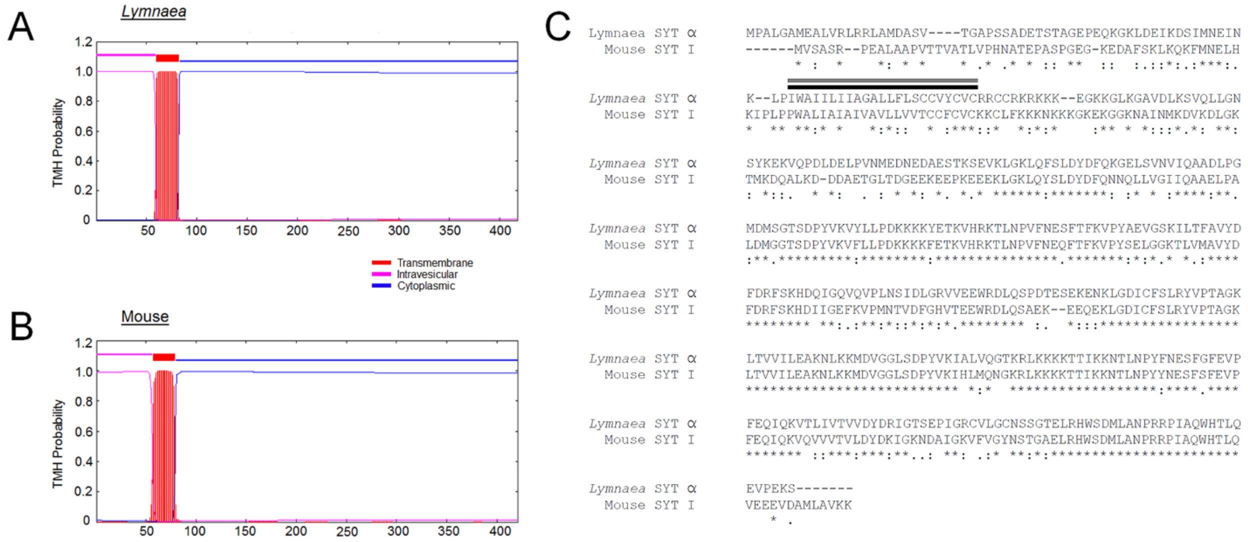

Supplementary Figure S6C: Prediction of *Lymnaea* synaptotagmin I C2B-α transmembrane domain

(**A-B**). Amino acid residue hydrophobicity plots predict the membrane topology and location of transmembrane helices in *Lymnaea* Syt I C2B-α (**A**) and mouse Syt I (**B**; accession no. D37792). Blue indicates predicted cytoplasmic residues, red indicates predicted transmembrane residues, pink indicates predicted intravesicular residues (TMHMM 2.0; CBS). (**C**). Protein sequence alignment of *Lymnaea* (top) and mouse (bottom) Syt I (Clustal Omega; EMBL-EBI). Grey bar indicates the predicted *Lymnaea* transmembrane domain, black bar indicates the predicted mouse transmembrane domain. The sequence identity of *Lymnaea* and mouse synaptotagmin I homologues is ~60%.

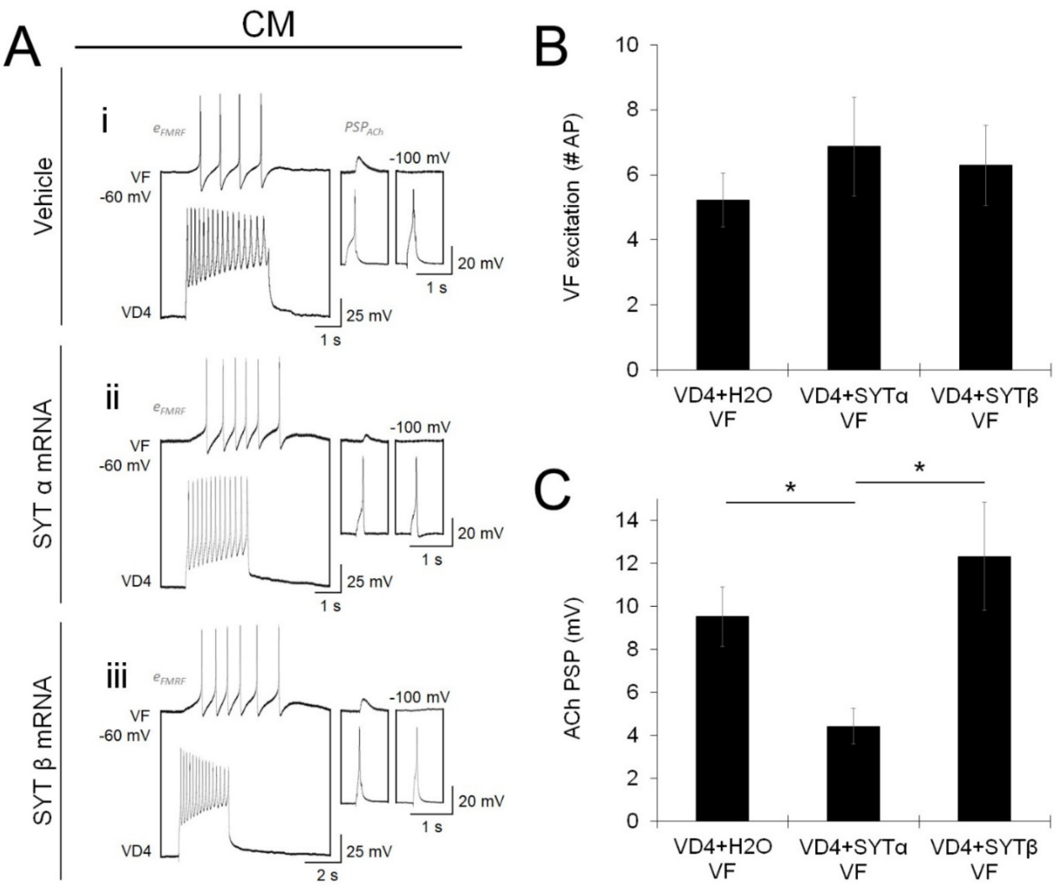

Supplementary Figure S6D: *Lymnaea* synaptotagmin homologues affect classical but not neuropeptide synaptic transmission

(**A**). Simultaneous recordings of VD4-VF neurons paired in CM, before and after the application of AChR antagonists (5 μM MLA, 10 μM TC, 20 μM TEA). VD4 was microinjected with H_2O (**i**; vehicle control), or synthetic *L*-Syt I C2B-α (**ii**) or *L*-Syt I C2B-β (**iii**) mRNA. Main panels show recordings made with AChR antagonists to isolate peptidergic transmission. Inserts show ACh-PSPs recorded in control conditions (left; CM control conditions; -100 mV holding potential) to assess cholinergic transmission. ACh-PSPs are inhibited by AChR antagonists (right). (**B**). Summary data, mean peptidergic synaptic response in VF measured in the presence of AChR antagonists, Syt I C2B-α or Syt I C2B-β overexpression does not influence FMRF neuropeptide release. N≥8. (5.22 ± 0.83; 6.87 ± 1.52; 6.29 ± 1.23 APs). *P*=0.661 (One-way ANOVA). (**C**). Summary data, mean ACh-PSP amplitudes of VD4-VF synapses, Syt I C2B-α overexpression attenuates ACh release. N≥10. (9.54 ± 1.39; 4.42 ± 0.82; 12.34 ± 2.51 mV). *F*=7.112; *P*=0.029 (Independent Samples Kruskal-Wallis test). *, *P*=0.030; *P*=0.014. Error bars, SEM.

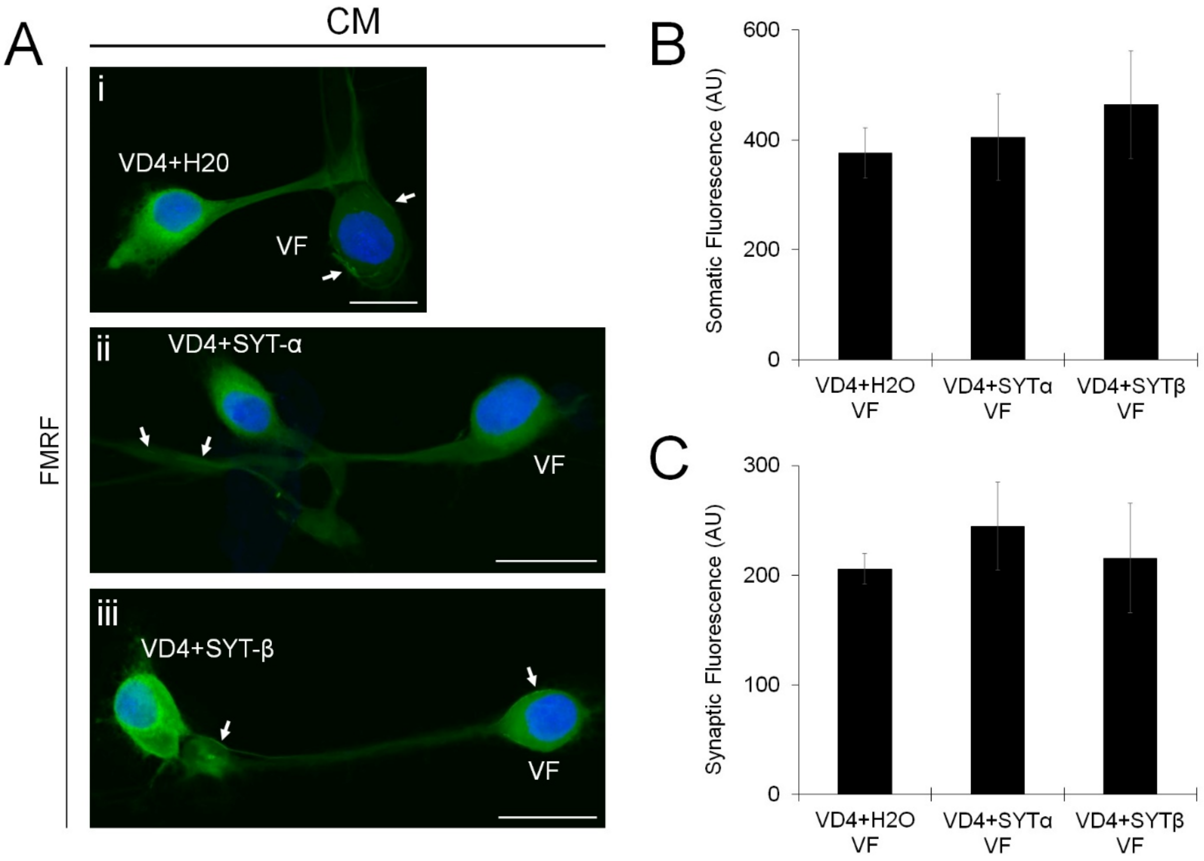

Supplementary Figure S6E: *Lymnaea* synaptotagmin homologues do not affect FMRF neuropeptide levels

(**A**). ICC labeling of FMRF neuropeptides in VD4 neurons microinjected with H_2O (**i**; vehicle control) or synthetic *L*-Syt I C2B-α (**ii**) or *L*-Syt I C2B-β (**iii**) mRNA, and paired with VF in CM. Scale bars, 50 µm. (**B**). Summary data, mean fluorescence intensity of somatic FMRF neuropeptides is not affected by Syt overexpression. N≥5. (376.42 ± 45.52; 405.38 ± 79.08; 464.59 ± 97.68). F=0.795; P=0.672 (Independent Samples Kruskal-Wallis test). (**C**). Summary data, mean fluorescence intensity of synaptic FMRF neuropeptides is not affected by Syt overexpression. N≥5. (205.75 ± 13.97; 244.79 ± 39.91; 215.65 ± 50.15). F=0.327; P=0.726 (One-way ANOVA).

SUPPLEMENTARY EXPERIMENTAL PROCEDURES

Table S10. *Lymnaea* Cloning Primers

Primer	Sequence
L-Syp 5’	GATGGGCCCATGGAGGCCGCCGGACAG
L-Syp 3’	GATGAATTCTCAAGCTGTGTGGGACTCTAC
L-Syt I 5’	GATCTCGAGATGCCTGCCCTG
L-Syt I 3’	GATGGATCCTTAGCTCTTCTCTG
L-Syp-mCherry 5’	GATGATGAATTCATGGAGGCCGCCGGACAG
L-Syp-mCherry 5’ SOE	CCAACACAGCCCCATTGCTGGGAATGTGTATGGGTCTGGGTCT GGGTCTGGGATGGTGAGCAAGGGCGAGGAGGATAACATGG
L-Syp-mCherry 3’ SOE	CCATGTTATCCTCCTCGCCCTTGCTCACCATCCCAGACCCAGAC CCAGACCCATACACATTCCCAGCAATGGGGCTGTGTTGG
L-Syp-mCherry 3’	GATGATGCGGCCGCTTACTTGTACAGCTCGTCCATGC

SUPPLEMENTARY REFERENCES

1 Cottrell, G. A. & Davies, N. W. Multiple receptor sites for a molluscan peptide (FMRFamide) and related peptides of Helix. *The Journal of physiology* 382, 51-68 (1987).

2 Wang, Z., Lange, A. B. & Orchard, I. Coupling of a single receptor to two different G proteins in the signal transduction of FMRFamide-related peptides. *Biochemical and biophysical research communications* 212, 531-538 (1995).

3 Skingsley, D. R. *et al.* A molecularly defined cardiorespiratory interneuron expressing SDPFLRFamide/GDPFLRFamide in the snail Lymnaea: monosynaptic connections and pharmacology. *Journal of neurophysiology* 69, 915-927 (1993).

4 Johnston, P. A., Jahn, R. & Sudhof, T. C. Transmembrane topography and evolutionary conservation of synaptophysin. *The Journal of biological chemistry* 264, 1268-1273 (1989).

5 Nakhost, A., Houeland, G., Blandford, V. E., Castellucci, V. F. & Sossin, W. S. Identification and characterization of a novel C2B splice variant of synaptotagmin I. *Journal of neurochemistry* 89, 354-363, doi:10.1111/j.1471-4159.2004.02325.x (2004).

6 Nakhost, A., Houeland, G., Castellucci, V. F. & Sossin, W. S. Differential regulation of transmitter release by alternatively spliced forms of synaptotagmin I. *The Journal of neuroscience : the official journal of the Society for Neuroscience* 23, 6238-6244 (2003).
